# Comprehensive analyses of the *annexin* (*ANN*) gene family in *Brassica rapa*, *Brassica oleracea* and *Brassica napus* reveals their roles in stress response

**DOI:** 10.1038/s41598-020-59953-w

**Published:** 2020-03-09

**Authors:** Xin He, Li Liao, Sai Xie, Min Yao, Pan Xie, Wei Liu, Yu Kang, Luyao Huang, Mei Wang, Lunwen Qian, Zhongsong Liu, Chunyun Guan, Mei Guan, Wei Hua

**Affiliations:** 1 Southern Regional Collaborative Innovation Center for Grain and Oil Crops in China, Hunan Agricultural University, Changsha, Hunan 410128 China; 2 Oil Crops Research, Hunan Agricultural University, Changsha, Hunan 410128 China; 3 Hunan Branch of National Oilseed Crops Improvement Center, Changsha, Hunan 410128 China; 4 Oil Crops Research Institute of the Chinese Academy of Agricultural Sciences, Key Laboratory of Biology and Genetic Improvement of Oil Crops, Ministry of Agriculture and Rural Affairs, Wuhan, 430062 China

**Keywords:** Plant hormones, Plant stress responses

## Abstract

Annexins (ANN) are a multigene, evolutionarily conserved family of calcium-dependent and phospholipid-binding proteins that play important roles in plant development and stress resistance. However, a systematic comprehensive analysis of *ANN* genes of Brassicaceae species (*Brassica rapa*, *Brassica oleracea*, and *Brassica napus*) has not yet been reported. In this study, we identified 13, 12, and 26 *ANN* genes in *B. rapa*, *B. oleracea*, and *B. napus*, respectively. About half of these genes were clustered on various chromosomes. Molecular evolutionary analysis showed that the *ANN* genes were highly conserved in Brassicaceae species. Transcriptome analysis showed that different group *ANN* members exhibited varied expression patterns in different tissues and under different (abiotic stress and hormones) treatments. Meanwhile, same group members from *Arabidopsis thaliana*, *B. rapa*, *B. oleracea*, and *B. napus* demonstrated conserved expression patterns in different tissues. The weighted gene coexpression network analysis (WGCNA) showed that *BnaANN* genes were induced by methyl jasmonate (MeJA) treatment and played important roles in jasmonate (JA) signaling and multiple stress response in *B. napus*.

## Introduction

Annexins (ANN) are a multigene, evolutionarily conserved family of calcium (Ca^2+^)-dependent and phospholipid-binding proteins present in plants, animals, and microorganisms^1,2^. ANN contain the characteristic annexin repeat and they regulate membrane dynamics, mediate Ca^2+^ sensing and signaling, link Ca^2+^ dynamics to cytoskeletal responses, and mediate immune or stress responses and signaling during plant growth and development^1,3^. A typical ANN contains four annexin repeats at the C-terminal region and a highly variable N-terminal region. Each annexin repeat usually contains a characteristic type II motif for Ca^2+^ binding^1,3^. The variable N-terminal region interacts with other proteins and is responsible for the functional diversity of ANN^4^.

Recent studies have identified the *ANN* gene family in *Arabidopsis thaliana* (8 genes), *Brassica rapa* (13), *Solanum lycopersicum* (9), *Solanum tuberosum* (9), *Oryza sativa* (10), *Triticum aestivum* (25), *Gossypium raimondii* (14), *Arachis hypogaea L*. (8), *Hordeum vulgare* (11), *Medicago truncatula* (10)*, Populus trichocarpa* (12)*, Vitis vinifera* (14)*, Carica papaya* (12)*, Glycine max* (22)*, Cochliobolus sativus* (11)*, Sorghum bicolor* (10)*, Zea mays* (12), *Brachypodium distachyon* (11), *Selaginella mollendorffii* (5), and *Physcomitrella patens* (7) via genome-wide analysis^2,5–11^.

Studies have shown that *ANN* gene family plays a significant role in plant development and plant protection during both abiotic and biotic stresses^1,3,12,13^. In *Arabidopsis*, two *ANN* genes (*AtANN1* and *AtANN4*) were regulated by abiotic stress, negatively regulated plant tolerance to drought, salinity, and heat stress, while *AtANN8* was positive regulated the plant abiotic tolerance^14–21^. Studies also demonstrated that *AtANN1* and *AtANN2* regulated root growth and development^20–22^. Downregulation of AtANN5 resulted in abnormal pollen grains and severe male sterility^23,24^. The rice annexin OsANN1 enhanced heat stress tolerance^25^, and OsANN3 positively regulated drought tolerance^26^. ZmANN33 and ZmANN35 enhanced chilling stress tolerance during germination of maize seeds^27^. *Medicago truncatula* annexin 1 regulated nodulation and mycorrhization in legume plants^28,29^. The tobacco annexin *Ntann12* was induced upon *Rhodoccocus fascians* infection^30,31^. The potato annexin STANN1 promoted drought tolerance^10^. The cotton annexin gene *GhAnn1* was induced by various phytohormones and abiotic stress, positively regulated drought and salt tolerance^32^. GhANN8b and phosphatase GhDsPTP3a proteins of cotton interacted with each other and regulated salt tolerance and calcium influx^33^. *GhAnn2* was induced by IAA and GA3, and *GhAnn2* downregulation inhibited cotton fiber elongation by modulating Ca^2+^ influx at the cell apex^8^. GhFAnnxA regulated fiber elongation and secondary cell wall biosynthesis^34^. AnxGb6 interacted with actin1 and regulated cotton fiber elongation^35^. Overexpression of cotton annexin gene *AnnGh3* increased trichome density and length in *Arabidopsis* leaf^36^. Overexpression of *Brassica juncea* annexin *AnnBj2* increased salt tolerance and abscisic acid (ABA) insensitivity in transgenic plants^37,38^. Ectopic expression of *B. juncea* annexin gene *BjAnn1* in tobacco and cotton enhanced tolerance to various abiotic stresses and fungal pathogen^39–41^. AnnBj3 promoted oxidative stress tolerance in plants^42^.

*Brassica napus* (genome AnAnCnCn) is an important oil crop worldwide, which was formed by recent allopolyploidy between ancestors of *Brassica rapa* (genome ArAr) and *Brassica oleracea* (genome CoCo)^43^. The production and quality of *B. napus* is greatly influence by adverse environmental conditions. Therefore, it is critical to improve stress tolerance in *B. napus* through the identification and use of genes involved in stress response. Although there are many studies on *ANN* genes in various plant species, *ANN* genes are yet to be characterized in *B. napus* and *B. oleracea*. In this study, we investigate the potential role of *ANN* genes in environmental stress response in Brassicaceae plants. Therefore, we identified the *ANN* genes of *B. napus*, *B. rapa* and *B. oleracea* and compared the gene structure, chromosomal location, evolutionary relationship, and expression pattern in different tissues and under different abiotic/biotic stresses and plant hormonal treatments. The findings of this study will provide a foundation for further studies on functional characterization of *ANN* genes of Brassicaceae plants under adverse environmental conditions.

## Results and Discussion

### Identification of ANN in *B. rape*, *B. oleracea* and *B. napus*

A total of 13 BrANN (*B. rapa* ANN), 12 BoANN (*B*. *oleracea* ANN), and 26 BnaANN (*B. napus* ANN) proteins were identified through BLASTP using 8 *Arabidopsis* ANN (AtANN) proteins (Table 1). All members were verified for the presence of annexin repeats using InterPro and Conserved Domain (CD)-search in NCBI. Brassicaceae species experienced an extra whole-genome triplication (WGT) event^44–46^, based on which approximately 24 and 48 *ANN* genes were expected in *B. rapa*/*B. oleracea* and *B. napus* genomes, respectively. However, only 13, 12, and 26 ANN genes were found in *B. rapa*, *B. oleracea*, and *B. napus*, respectively (Table 1). In *B. napus*, the number of genes in the An-subgenome (12) and Cn-subgenome (14) was almost the same as that in their diploid progenitors *B. rapa* and *B. oleracea* (Table 1). These results indicate the loss of about half of *ANN* genes after the Brassicaceae WGT in *B. rapa* and *B. oleracea*. However, most of the duplicated *ANN* genes were retained after the whole-genome duplication (WGD) event in *B. napus*. WGD event of gene family appears to be a widespread phenomenon, such as the *auxin response factor* (*ARF*)^47^, *Auxin/Indoleacetic acid* (*Aux/IAA*)^48^, *glutathione transferases* (*GST*)^49^, *BRI1-EMS-SUPPRESSOR1* (*BES1*)^50^, *Heat stress transcription factors* (*Hsfs*)^51,52^, *GRAS*^53^ family genes in diploid and allopolyploid Brassicaceae and *Calcium-dependent protein kinases* (*CPK*)^54^, *Jasmonate ZIM-domain* (*JAZ*)^55^ and *Nuclear factor YB* (*NF-YB*)^56^ in diploid and allopolyploid *Gossypium* species (*G. raimondii*: DD genome; *G. arboretum*: AA genome; *G. hirsutum*: AADD genome).

**Table 1:** List of *ANN* genes identified in *Arabidopsis*, *B. rape*, *B. oleracea* and *B. napus*.

*Arabidopsis*	homologous gene in *B. rape*/*B. oleracea/B. napus*	Gene ID	Gene name	CDS (bp)	AA	pI	Mw (kD)	Introns	Exons	Annexin repeats	Predicted subcellular localization	Chromosome location
*AT5G10220 (ANN6)*	*B. rape*	*Bra009049*	*BrANN6*	957	318	6.73	36.43	3	4	4	Cytoplasmic	A10:15026227-15027937
	*B. oleracea*	*Bo9g172340*	*BoANN6*	957	318	7.69	36.55	3	4	4	Cytoplasmic	C09:50988876-50990648
	*B. napus*	*BnaA10g22020D*	*BnaANN6A*	957	318	6.43	36.41	3	4	4	Cytoplasmic	chrA10:14972075.14973785
		*BnaC09g46410D*	*BnaANN6C*	957	318	7.69	36.55	3	4	4	Cytoplasmic	chrC09:46266111.46267883
*AT5G10230 (ANN7)*	*B. rape*	*Bra009048*	*BrANN7*	951	316	6.43	36.38	3	4	4	Cytoplasmic	A10:15023513-15025336
	*B. oleracea*	*Bo9g172330*	*BoANN7*	552	183	8.67	21.10	1	2	2	NONE	C09:50986649-50987380
	*B. napus*	*BnaA10g22010D*	*BnaANN7A*	951	316	6.73	36.43	3	4	4	Cytoplasmic	chrA10:14969359.14971179
		*BnaC09g46400D*	*BnaANN7C*	636	211	6.85	24.54	3	4	2	NONE	chrC09:46263425.46264615
*AT5G65020 (ANN2)*	*B. rape*	*Bra031890*	*BrANN2-1*	951	316	6.1	36.16	4	5	4	Cytoplasmic	A02:27252010-27253638
	*B. oleracea*	*Bo2g166530*	*BoANN2-1*	951	316	6.1	36.15	4	5	4	Cytoplasmic	C02:52272616-52275200
	*B. napus*	*BnaAnng37420D*	*BnaANN2A-1*	690	229	6.46	25.35	2	3	3	NONE	chrAnn_random:42372910.42373846
		*BnaC02g43450D*	*BnaANN2C-1*	951	316	6.1	36.15	4	5	4	Cytoplasmic	chrC02:45624871.45627387
	*B. rape*	*Bra024346*	*BrANN2-2*	951	316	5.97	36.00	3	4	4	Cytoplasmic	A06:15094250-15096367
	*B. oleracea*	*no*										
	*B. napus*	*BnaA06g23960D*	*BnaANN2A-2*	714	237	7.02	26.91	2	3	3	Cytoplasmic	chrA06:16571045.16572233
		*BnaC03g49290D*	*BnaANN2C-2*	951	316	5.97	36.00	3	4	4	Cytoplasmic	chrC03:34204881.34206680
*AT1G35720 (ANN1)*	*B. rape*	*Bra039578*	*BrANN1-3*	789	265	6.2	30.43	4	5	3	NONE	Scaffold000169:141815-143464
	*B. oleracea*	*Bo6g043900*	*BoANN1-3*	960	319	5.27	36.39	5	6	4	Cytoplasmic	C06:11348510-11350276
	*B. napus*	*no*										
		*BnaC06g08410D*	*BnaANN1C-3*	657	218	6.15	25.41	3	4	2 or 3	NONE	chrC06:9571468.9572381
	*B. rape*	*Bra036764*	*BrANN1-1*	954	317	5.42	36.18	4	5	4	Cytoplasmic	A08:7174909-7176387
	*B. oleracea*	*Bo8g025760*	*BoANN1-1*	954	317	5.36	36.19	4	5	4	Cytoplasmic	C08:7217833-7219309
	*B. napus*	*BnaA08g06260D*	*BnaANN1A-1*	954	317	5.29	36.16	4	5	4	Cytoplasmic	chrA08:6213624.6215101
		*BnaC08g06690D*	*BnaANN1C-1*	954	317	5.36	36.19	4	5	4	Cytoplasmic	chrC08:9209378.9210853
	*B. rape*	*Bra034402*	*BrANN1-2*	978	325	5.17	37.07	4	5	4	Cytoplasmic	A05:13457651-13459433
	*B. oleracea*	*Bo5g083900*	*BoANN1-2*	954	317	5.34	36.10	4	5	4	Cytoplasmic	C05:27113881-27115646
	*B. napus*	*BnaAnng04520D*	*BnaANN1A-2*	954	317	5.34	36.10	4	5	4	Cytoplasmic	chrAnn_random:5214141.5215923
		*BnaC05g27530D*	*BnaANN1C-2*	954	317	5.27	36.10	4	5	4	Cytoplasmic	chrC05:24720391.24722156
*AT5G12380 (ANN8)*	*B. rape*	*no*										
	*B. oleracea*	*Bo1g039570*	*BoANN8-1*	474	157	9.12	17.81	2	3	1	NONE	C01:12051544-12052464
	*B. napus*	*no*										
		*BnaC01g16910D*	*BnaANN8C-1*	474	157	9.12	17.84	2	3	1	NONE	chrC01:11572050.11575202
	*B. rape*	*Bra008892*	*BrANN8-2*	948	315	6.57	35.64	5	6	4	Cytoplasmic	A10:14320354-14321828
	*B. oleracea*	*no*										
	*B. napus*	*BnaA10g20320D*	*BnaANN8A-2*	948	315	6.57	35.58	5	6	4	Cytoplasmic	chrA10:14257731.14259203
		*BnaC09g44350D*	*BnaANN8C-2*	948	315	6.8	35.63	5	6	4	Cytoplasmic	chrC09:45227676.45229144
*AT2G38760 (ANN3)*	*B. rape*	*Bra017102*	*BrANN3-1*	960	319	5.32	35.81	5	6	4	Cytoplasmic	A04:16602953-16604489
	*B. oleracea*	*Bo4g187790*	*BoANN3-1*	960	319	5.33	35.84	5	6	4	Cytoplasmic	C04:50339028-50340578
	*B. napus*	*BnaA04g22190D*	*BnaANN3A-1*	960	319	5.16	35.80	5	6	4	Cytoplasmic	chrA04:16761274.16762791
		*BnaC04g45920D*	*BnaANN3C-1*	960	319	5.42	35.76	5	6	4	Cytoplasmic	chrC04:45511897.45513442
	*B. rape*	*Bra000091*	*BrANN3-2*	960	319	5.86	35.96	5	6	4	NONE	A03:9218397-9219801
	*B. oleracea*	*Bo3g032770*	*BoANN3-2*	960	319	5.71	36.07	5	6	4	NONE	C03:12611466-12613000
	*B. napus*	*BnaA03g18080D*	*BnaANN3A-2*	960	319	6.05	35.98	5	6	4	NONE	chrA03:8500385.8501854
		*BnaC03g21600D*	*BnaANN3C-2*	960	319	6.05	36.03	5	6	4	NONE	chrC03:11687229.11688767
*AT1G68090 (ANN5)*	*B. rape*	*Bra033961*	*BrANN5*	951	316	9.56	35.99	5	6	4	Cytoplasmic	A02:10580974-10582455
	*B. oleracea*	*Bo2g057430*	*BoANN5*	951	316	9.56	35.99	5	6	4	Cytoplasmic	C02:16800135-16801609
	*B. napus*	*BnaA02g13560D*	*BnaANN5A*	951	316	9.56	35.99	5	6	4	Cytoplasmic	chrA02:7447649.7449167
		*BnaC02g45910D*	*BnaANN5C*	951	316	9.56	35.99	5	6	4	Cytoplasmic	chrC02_random:1724358.1725824
*AT2G38750 (ANN4)*	*B. rape*	*Bra000090*	*BrANN4-1*	948	315	7.25	35.52	5	6	2 or 3	NONE	A03:9214531-9216318
	*B. oleracea*	*Bo3g032760*	*BoANN4-1*	948	315	6.93	35.52	5	6	2 or 3	NONE	C03:12607451-12609054
	*B. napus*	*BnaA03g18070D*	*BnaANN4A-1*	948	315	7.25	35.52	5	6	3 or 3	NONE	chrA03:8495908.8497930
		*BnaC03g21590D*	*BnaANN4C-1*	948	315	6.93	35.52	5	6	2 or 3	NONE	chrC03:11679141.11681030
	*B. rape*	*Bra017103*	*BrANN4-2*	963	320	7.7	36.28	5	6	2 or 3	NONE	A04:16596347-16598662
	*B. oleracea*	*Bo4g187780*	*BoANN4-2*	963	320	8.44	36.32	5	6	2 or 3	NONE	C04:50333925-50335934
	*B. napus*	*BnaA04g22180D*	*BnaANN4A-2*	963	320	7.7	36.28	5	6	3 or 3	NONE	chrA04:16754601.16756932
		*BnaC04g45910D*	*BnaANN4C-2*	963	320	8.44	36.35	5	6	2 or 3	NONE	chrC04:45506797.45508806

Among the 51 *Brassica ANN* genes, 35 were the typical *ANN*, which encoded proteins ranging from 315–325 amino acids (AA) and contained four annexin repeats. All eight *ANN* members (315–320 AA) homologous to *AtANN4* (*AT2G38750*) contained 2–3 annexin repeats, as same as *AtANN4*. While two other *ANN* members (157 AA) contained only a single annexin repeat and six members (183–265 AA) contained 2–3 annexin repeats (Table 1), they may were the truncated mutated duplications.

### Phylogenetic and structural analysis of *ANN*

**Figure 1:**
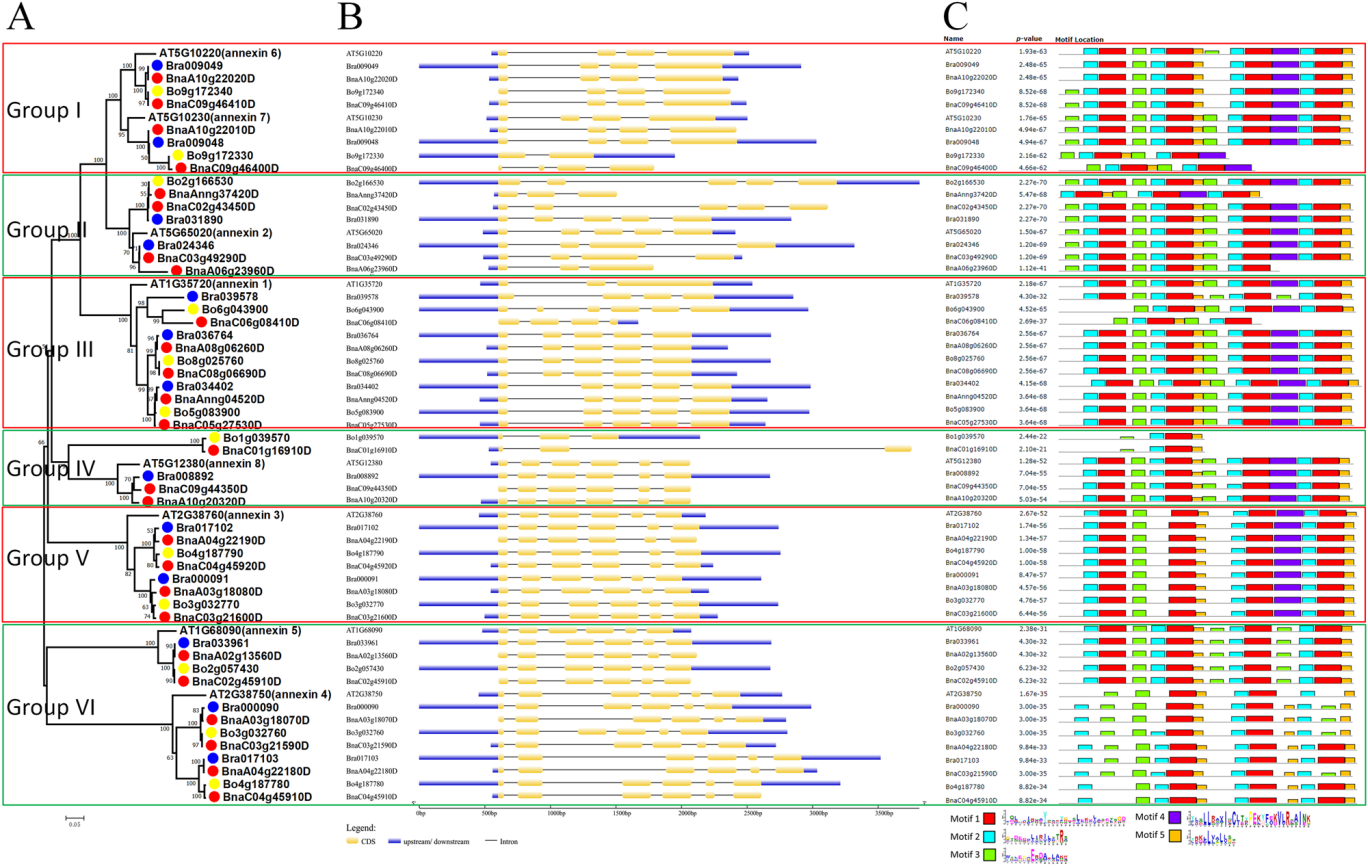
Phylogenetic tree (**A**), gene structure (**B**), and gene motifs (**C**) of ANN of *Arabidopsis*, *B. rapa*, *B. oleracea*, and *B. napus*. Neighbor-joining phylogenetic tree showing the relationship among 13 *B. rapa* (blue dots), 12 *B. oleracea* (yellow dots), 26 *B. napus* (red dots), and 8 *Arabidopsis* ANN proteins (**A**). The resulting six groups are labeled (Group I-VI). Orange boxes, black lines, and blue boxes indicate exons, introns, and untranslated regions, respectively (**B**). Five motifs in BnaSAP proteins were identified by MEME tools (**C**).

A phylogenetic tree (Fig. 1A) was generated using the sequences of 59 ANN proteins from *B. rapa*, *B. oleracea*, *B. napus*, and *Arabidopsis* (Fig. S1). These ANN proteins were divided into six groups. All eight *AtANN* were found to have orthologous genes in *B. rapa*, *B. oleracea*, and *B. napus* (Fig. 1A). Twelve pairs of *BnaANN* were found in the corresponding *B. napus* An- and Cn-homoeologous chromosomes, and ten pairs of them had homologous genes both in *B. rapa* and *B. oleracea*. Meanwhile, two pairs (*BnaC03g49290D/BnaA06g23960D* and *BnaC09g44350D/BnaA10g20320D*) only had homologous genes in *B. rapa*. All 12 *BoANN* genes were found to have homologous genes in the Cn-subgenome of *B. napus*, while one *BrANN* (*Bra039578*) had no homologous gene in An-subgenome of *B. napus* (Table 1 and Fig. 1A).

Gene structure analysis revealed that majority of the homologous *ANN* gene pairs had same gene structure (Fig. 1B). There were five introns in group IV/V/VI members, expect for two truncated mutant genes (*Bo1g039570* and *BnaC01g16910D*) (Fig. 1B). This finding indicates that the *ANN* genes are conserved in Brassicaceae species, possibly due to their importance in plant growth and productivity.

A typical ANN protein contains four annexin repeats, each approximately 70 amino acids long^1,3^. Annexin repeat usually contain a characteristic type II motif for binding calcium ions with the sequence GxGT-[38 residues]-D/E^3^. MEME analysis showed that 42 ANN proteins contained four annexin repeats (Fig. 1C). Motif1 was the core sequence of all the four annexin repeats, and motif4 was only found in the third annexin repeat in group I–V, while motif5 was the core sequence close to the C-terminal of Motif1 in the second and fourth annexin repeats (Fig. 1C).

According to the gene structure and motif analysis, the missing parts of the truncated mutant members were readily apparent. Both the first and fourth annexin repeats were absent in Bo9g172330 and BnaC09g46400D, and the first annexin repeat was absent in BnaAnng37420D. Bo1g039570 and BnaC01g16910D had only the second annexin repeat at the C-terminal (80–159 AA), and the core sequence of annexin repeat was not detected at the N-terminal (1–79 AA). It is similar in the N-terminal of Bo6g043900, BnaC06g08410D, and AtANN4 homologues (Fig. 1B,C).

### Chromosomal location and synteny analysis of *ANN* of *B. rapa*, *B. oleracea*, and *B. napus*

**Figure 2:**
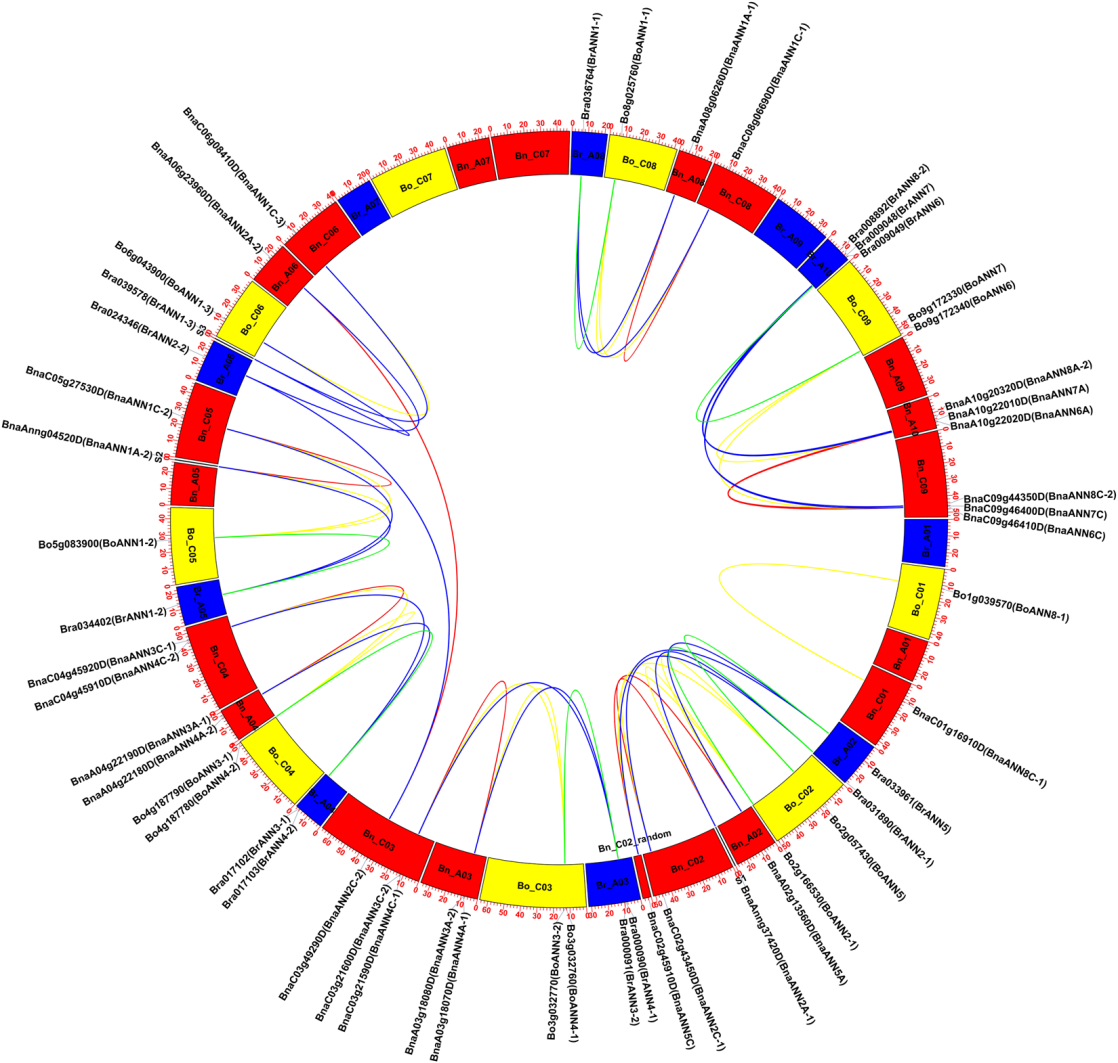
The synteny analysis of *ANN* genes in the *B. rapa, B. oleracea* and *B. napus* chromosomes. Br_A (*B. rapa* chromosomes): yellow trapezoid; Bo_C (*B. oleracea* chromosomes): blue trapezoid; Bn_A (*B. napus* An-subgenome chromosomes) and Bn_C (*B. napus* Cn-subgenome chromosomes): red trapezoid; Bn_C02_Random means genes were randomly distributed to *B. napus* Cn-subgenome chromosome C02. S1 and S2 are two unanchored scaffolds from *B. napus*; S3 is an unanchored scaffold (Scaffold000169) from *B. rapa*. The orthologous and paralogous *ANN* genes were mapped onto the chromosomes and linked by each other. Yellow lines linked two syntenic *ANN* genes from *B. rapa* and *B. napus*; Blue lines linked two syntenic *ANN* genes from *B. oleracea* and *B. napus*; Green lines linked two syntenic *ANN* genes from *B. rapa* and *B. oleracea*; Red lines linked two syntenic ANN genes from *B. napus* An- and Cn-subgenome.

As showed in Fig. 2, the distribution of *BnaANN* in An- and Cn-subgenome was nearly even with 12 *ANN* genes from the An-subgenome and 14 from the Cn-subgenome. However, the ANN genes’ distribution was uneven on each chromosome. Three pair (2 genes/pair) of *ANN* genes from the An-subgenome were repeated in tandem on chromosome Bn_A03, Bn_A04, and Bn_A10 (Fig. 2); and three pair (2 genes/pair) of *ANN* genes from the Cn-subgenome were repeated in tandem on chromosome Bn_C03, Bn_C04, and Bn_C09 (Fig. 2C). *B. napus* genome analysis showed that the An- and Cn-subgenome were largely collinear to the corresponding diploid Ar and Co genomes^43,57^. Most of the An-Ar and Cn-Co orthologous gene pairs demonstrated similar chromosomal locations. The distribution of *ANN* genes in *B. rapa* and *B. oleracea* were similar to the distribution of the orthologous *BnaANN* genes in the *B. napus* An-subgenome and Cn-subgenome, respectively (Fig. 2). Two *BnaANN* (*BnaAnng04520D* and *BnaAnng37420D*) and one *BrANN* (*Bra039578*) genes were located on the unanchored scaffolds that were not mapped to a specific chromosome (Fig. 2). The sequence and phylogenetic analyses revealed *BnaAnng04520D-Bra034402* and *BnaAnng37420D-Bra031890* as two An-Ar orthologous gene pairs. Based on this, we predicate Bn_A02 and Bn_A05 as the true chromosomal locations of *BnaAnng04520D* and *BnaAnng37420D*, respectively. *BnaANN* (*BnaC03g49290D* and *BnaC09g44350D*) had no orthologous genes in *B. oleracea* (Fig. 2), though they had homologous genes in An-subgenome. These findings indicate that duplication of *BnaA06g23960D* and *BnaA10g20320D* led to the formation of *BnaC03g49290D* and *BnaC09g44350D*, respectively. Analysis of the synteny among An-subgenome and Cn-subgenome showed high collinearity between Bn_A01-Bn_C01, A02-C02, A03-C03, A04-C04, A05-C05, A06-C06, A07-C07, A08-C08, A09-C09, and A10-C09, and 83.7% orthologous gene pairs between *B. rapa* and *B. oleracea* were retained as homologous gene pairs in *B. napus*^43,57^. 90.9% *ANN* gene pairs (10/11 pairs) between *B. rapa* and *B. oleracea* were retained as homologous gene pairs between *B. napus* An-chromosomes and Cn-chromosomes (Fig. 2).

There were two tandem pairs (*AtANN3/4* and *AtANN6/7*) on chromosome 2 and chromosome 5 in *Arabidopsis*, respectively^58^. *Bra009048/Bra009049*, *Bo9g172330/Bo9g172340*, *BnaA10g22010D/BnaA10g22020D*, and *BnaC09g46400D/BnaC09g46410D* were homologous to *AtANN6/7* tandem pair in *B. rapa*, *B. olearcea*, *B. napus* An-subgenome and Cn-subgenome, respectively. We identified two tandem pairs each homologous to *AtANN3/4* in *B. rapa* (Br_A03 and Br_A04), *B. olearcea* (Bo_C03 and Bo_C04), *B. napus* An-subgenome (Bn_A03 and Bn_A04), and Cn-subgenome (Bn_C03 and Bn_C04) (Fig. 2). *AtANN8* (*AT5G12380*) was located near the *AtANN6/7* tandem pair on chromosome 5 in *Arabidopsis*^58^. Correspondingly, there was a gene homologous to *AtANN8* located near the tandem pair homologous to *AtANN6/7* in *B. rapa* (Br_A10), *B. napus* An-subgenome (Bn_A10) and Cn-subgenome (Bn_C09) (Fig. 2). There was no gene homologous to *AtANN8* in *B. olearcea* (Bo_C09). Instead, we found a truncated mutated gene (*Bo1g039570*) homologous to *AtANN8* in *B. olearcea* (Bo_C01). Meanwhile, a truncated mutated gene (*BnaC01g016910D*) was homologous to *Bo1g039570* in *B. napus* Cn-subgenome (Bn_C01) (Fig. 2). *Bra039578* had no homologous gene in An-subgenome of *B. napus*. These findings suggest that majority of the *ANN* genes are conserved in Brassicaceae species, only a few *ANN* genes are missing or duplicating in *B. napus*.

To better understand the evolutionary constraints acting on the *ANN* gene family, we estimated the number of nonsynonymous substitutions per nonsynonymous site (*Ka*), the number of synonymous substitutions per synonymous site (*Ks*), and the *Ka/Ks* ratio. *Ka/Ks* value <1 indicates that a gene pair has experienced purifying selection; *Ka/Ks*>1 indicates positive selection; and *Ka/Ks* = 1 indicates neutral selection^59^. The *Ka/Ks* ratio was <1 for majority of the ANN collinear gene pairs (209/210), except for the gene pair *Bra024346/BnaA06g23960D* (*Ka/Ks* > 1) (Table S1). These results indicate that majority of genes experienced purifying selection, whereas *Bra024346* and *BnaA06g23960D* experienced positive selection.

### Expression profile of *ANN* genes in different tissues

**Figure 3:**
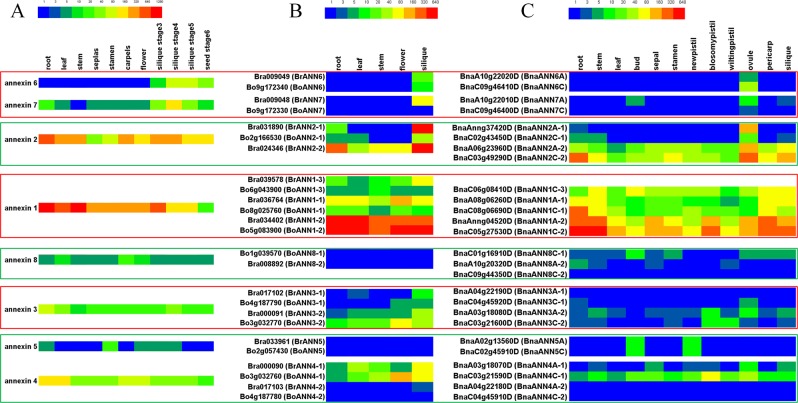
Heat map showing expression of *ANN* genes in different tissues at different developmental stages of *Arabidopsis* (**A**), *B. rapa* (**B**), *B. oleareca* (**B**), and *B. napus* (**C**). Coloured rectangles indicate the gene FPKM values.

*ANN* genes exhibit tissue-specific expression, which is usually consistent with their substantially differentiated functions^14,16–18,22,23,58^. We investigated the expression of all *ANN* genes in different tissues of *Arabidopsis*, *B. rapa*, *B. olearcea*, and *B. napus* based on the *Arabidopsis* eFP Browser data (http://bar.utoronto.ca/efp/cgi-bin/efpWeb.cgi) and RNA-Seq data (*B. rapa*: GSE43245; *B. oleareaca*: GSE42891; *B. napus*: PRJNA394926) (Table S2)^57,60,61^. The *ANN* genes were expressed across different vegetative and reproductive organs during different developmental stages of the four species (Fig. 3). In general, the *ANN* expression pattern was different between groups; however, expression pattern was very similar within a group in the four plant species.

Group I (*ANN6/7*) members showed expression in young siliques (ovules) and seeds, which indicate their importance in ovule and seed development in Brassicaceae plants. However, two truncated mutated members (*Bo9g172330* and *BnaC09g46400D*) homologous to *ANN7* were at low abundance expression levels (Fig. 3). Unlike *Bo9g172330* and *BnaC09g46400D*, other five truncated mutated members (*BnaAnng37420D*, *BnaA06g23960*, *BnaC06g08410D*, *Bo1g039570* and *BnaC01g16910D*) have a similar expression level to their homologous genes which have complete gene structure (Figs. 1 and 3). So, truncated mutated gene structures may decrease their own genes’ expression level, but not always. The expression levels of group 2 (*ANN2*) members were highest in roots and young siliques (ovules), while that of group 3 (*ANN1*) members were higher in roots, stems, and young siliques (pericarps) (Fig. 3). These expression levels are consistent with the role of *AtANN1* and *AtANN2* in root growth and development^20–22^. It was indicated that *ANN1/2* regulates the development of young siliques and seeds. We detected low level of expression for group 4 (*ANN8*) members. *AtANN5*, which regulates pollen development^23,24^, showed specific expression in mature pollen. The *B. napus* genes homologous to *AtANN5* were mainly expressed in buds and new pistils. The genes homologous to *AtANN3* and *AtANN4* demonstrated similar expression pattern. Both genes were expressed in flowers and young siliques (ovules), though they belong to group IV and VI, respectively (Fig. 3). All these indicated that *ANN* genes may be involved in various developmental processes with different functions. In *Arabidopsis*, *AtANN3* and *AtANN4* had similar expression pattern because they share a 5′ promoter region (2654 bp)^58^. In *B. rapa, Bra000090* and *Bra000091* share a 5′ promoter region (2079 bp), while in *B. oleareca*, *Bo3g032760* and *Bo3g032770* share a 5′ promoter region (2412 bp); In *B*. *napus*, *BnaA03g18070D* and *BnaA03g18080D* share a 5′ promoter region (2455 bp) and *BnaC03g21590D* and *BnaC03g21600D* share a 5′ promoter region (6199 bp). They were homologous to *AtANN3*/*AtANN4* pair, and had similar expression pattern. But another gene pairs (*Bra017102/Bra017103*, *Bo4g187790/Bo4g187780*, *BnaA04g22190D/BnaA04g22180D*, and *BnaC04g45920D/BnaC04g45910D*) homologous to *AtANN3*/*AtANN4* pair were at low abundance expression levels (Fig. 3B,C). All the results suggested that there were gene duplications, gene expression pattern differentiations and subsequent functional diversifications in *ANN* family genes in Brassicaceae species, and the functions of homologs of a given group *ANN* genes might be redundant as they share similar expression patterns.

### Expression pattern of *ANN* genes in response to abiotic stress and hormonal treatment

**Figure 4:**
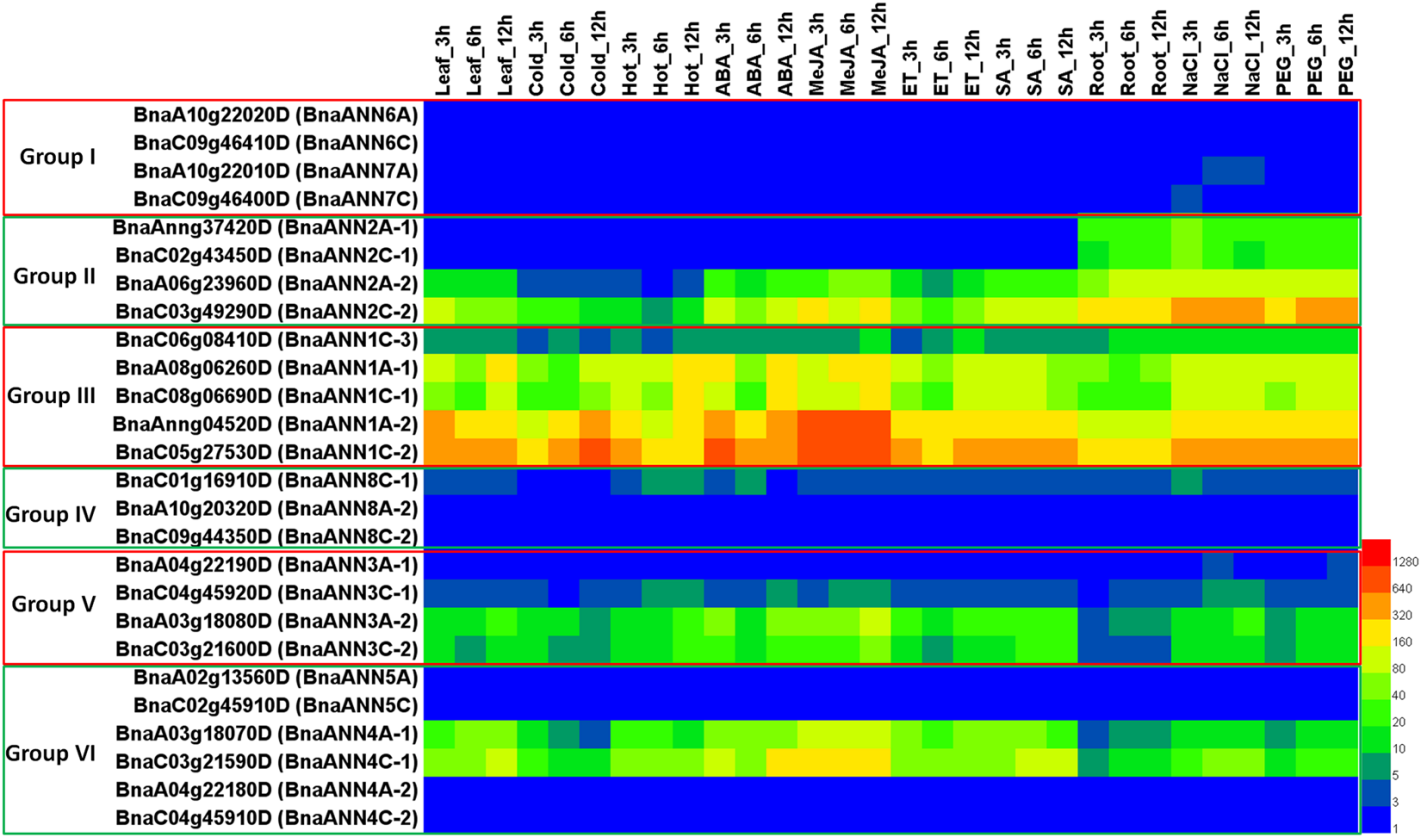
Expression of *BnaANN* under abiotic stress and plant hormone treatments. Leaf: untreated leaves; Cold: leaves treated with 4 °C; Hot: leaves treated with 40 °C; ABA: leaves treated with 100 μM abscisic acid; MeJA: leaves treated with 100 μM methyl jasmonate; ETH: leaves treated with 10 μg/ml ethephon; SA: leaves treated with 1.0 mM salicylic acid. Root: untreated roots; NaCl: roots treated with 200 mM NaCl; PEG: roots treated with 20% polyethylene glycol 6000. Coloured rectangles indicate TPM values.

Accumulating evidence from various plant species has shown the regulation of *ANN* genes in response to abiotic stress and hormonal treatment^5–7,9,58^. To examine the expression pattern of *BnaANN* genes under various abiotic stress conditions and hormonal treatments, we utilized the data on transcriptional profiling (Table S3). As shown in Fig. 4, most of the expressed *BnaANN* genes in group II/III/V/VI/were up-regulated under salinity and PEG stress in roots and MeJA treatment in leaves. *BnaA06g23960*, *BnaA03g18070D* and *BnaC03g21590D* were down-regulated under cold stress, whereas *BnaAnng04520D* and *BnaC05g27530D* were up-regulated under cold stress at 12 hours point (Fig. 4).

**Figure 5:**
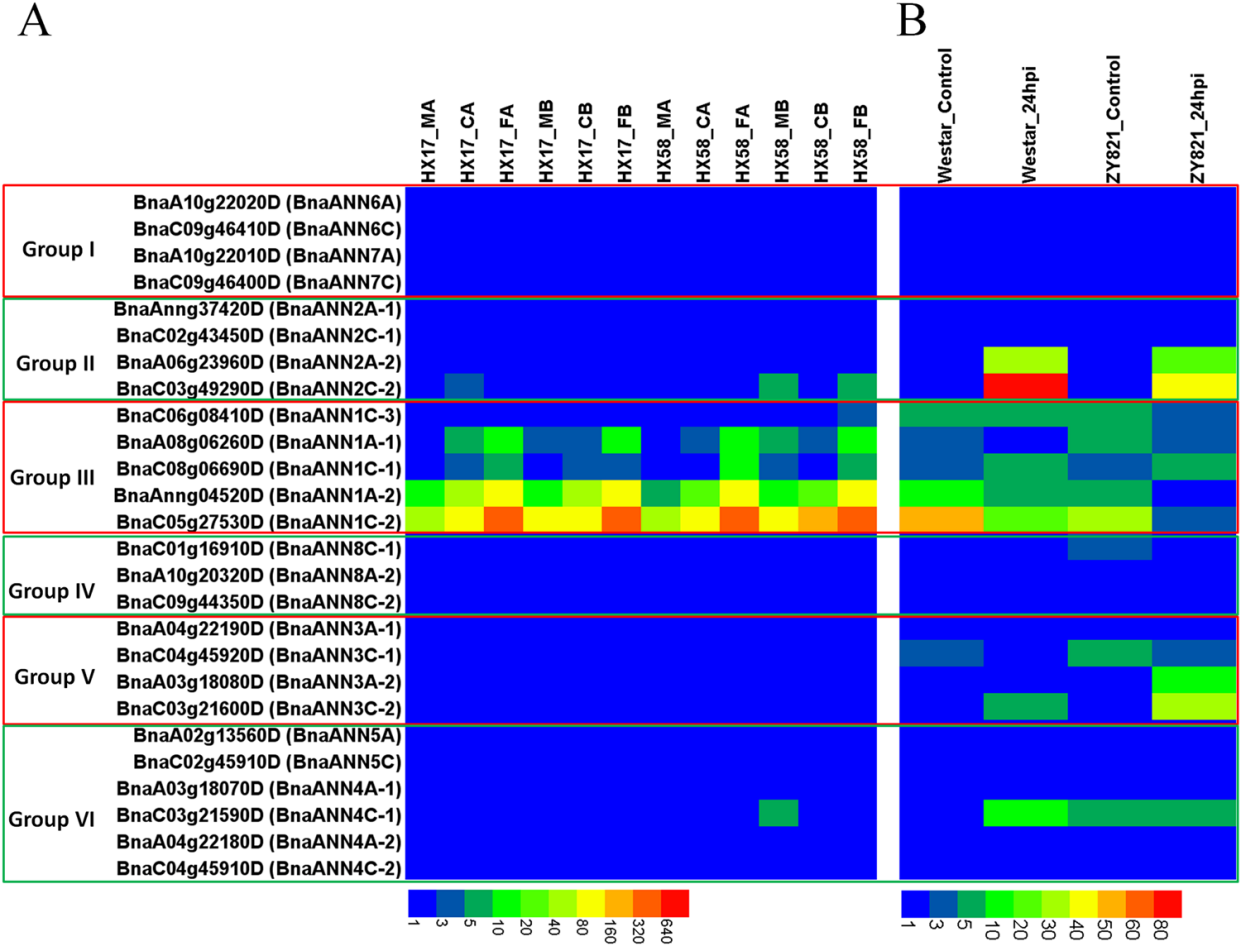
Expression profile of *BnaANN* under different low-temperature treatments in two early-maturing semi-winter *B. napus* varieties HX17 and HX58 (**A**) and in susceptible (Westar) and tolerant (ZY821) genotypes of *B. napus* infected with *Sclerotinia sclerotiorum* (**B**). MA: untreated leaves of 6-weeks-old seedlings; CA: leaves of 6-weeks-old seedlings treated with cold acclimation (4 °C for two weeks) and then treated 4 °C for 12 hours; FA: leaves of 6-weeks-old seedlings treated with cold acclimation (4 °C for two weeks) and then treated −4 °C for 12 hours; MB: untreated leaves of 6-weeks-old seedlings; CB: leaves of 6-weeks-old seedlings treated with 4 °C for 12 hours; FB: leaves of 6-weeks-old seedlings treated with −4 °C for 12 hours. Coloured rectangles indicate the gene FPKM values.

*B. napus* is a winter biennial crop with excellent tolerance to low-temperature stress during vegetative stage. The response mechanisms are different under chilling and freezing temperatures, as well as cold shock and cold acclimation in plants^62,63^. Based on the transcriptional profiling of early-maturing, cultivated *B. napus* varieties under different low-temperature treatments with or without cold acclimation (GSE129220: https://www.ncbi.nlm.nih.gov/geo/query/acc.cgi?acc=GSE129220) (Table S4)^64^, transcriptome analysis revealed that group III *BnaANN* were induced slightly by chilling stress, and were up-regulated by freezing stress strongly, regardless of cold acclimation (Fig. 5A). This finding indicates that group III *BnaANN* genes play important roles in freezing stress in *B. napus*.

*Sclerotinia sclerotiorum* is a hemibiotroph pathogen with a wide host range. It is the causative agent of stem rot, one of the most devastating diseases of *B. napus*^65,66^. Previous studies have shown the role of JA signaling in plant resistance to hemibiotroph pathogens^67–70^. The transcriptional profiling of *B. napus* susceptible (Westar) and tolerant (ZY821) genotypes infected with *S. sclerotiorum* (GSE81545: https://www.ncbi.nlm.nih.gov/geo/query/acc.cgi?acc=GSE81545) (Table S5) showed that the group II *BnaANN* were induced by *S. sclerotiorum* infection, and the expression level in the susceptible genotype (Westar) was more than that in the tolerant (ZY821) genotype; some members from group III and group V *BnaANN* were induced, while some members were repressed by *S. sclerotiorum* infection (Fig. 5B). These findings indicate a complex response mechanism and the role of some *BnaANN* in *B. napus* response to *S. sclerotiorum*.

**Figure 6:**
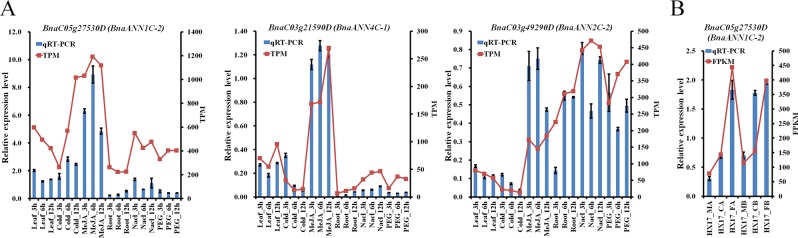
qRT-PCR analysis of three *BnaANN* genes under abiotic stress and hormone treatments. The relative qRT-PCR expression level (blue bar) is shown on the left y-axis. The RNA-Seq TPM/FPKM values (red line) are shown on the right y-axis. *BnaActin* (*BnaC05g34300D*) was used as the endogenous reference gene. The relative transcript levels were averaged over the three technical replicates.

To validate the results on transcriptional profiling, we performed a qRT-PCR to detect the transcript levels of three genes (*BnaC03g49290D*, *BnaC05g27530D*, and *BnaC03g21590D*) from group II/III/VI in the roots challenged with salt and PEG and in the leaves treated with cold and MeJA. The expression pattern (of these *BnaANN* genes) was consistent with the RNA-Seq data (Figs. 4–6). All three *BnaANN* genes were induced under salinity and PEG stress in roots and induced by MeJA in leaves (Fig. 6A). *BnaC03g49290D* and *BnaC03g21590D* were repressed by cold treatment (Fig. 6A). *BnaC05g27530D* was significantly upregulated under freezing stress, with or without cold acclimation (Fig. 6B). In *B. rapa*, *Bra034402* (gene to homologous *BnaC05g27530D*) was strongly induced by hormone and stress treatments^11^. All these results indicated the role of these three genes in multiple abiotic stress response and JA signaling response in *B. napus*.

### Weighted gene co-expression network analysis (WGCNA) of *BnaANN* in response to environmental stress

**Figure 7:**
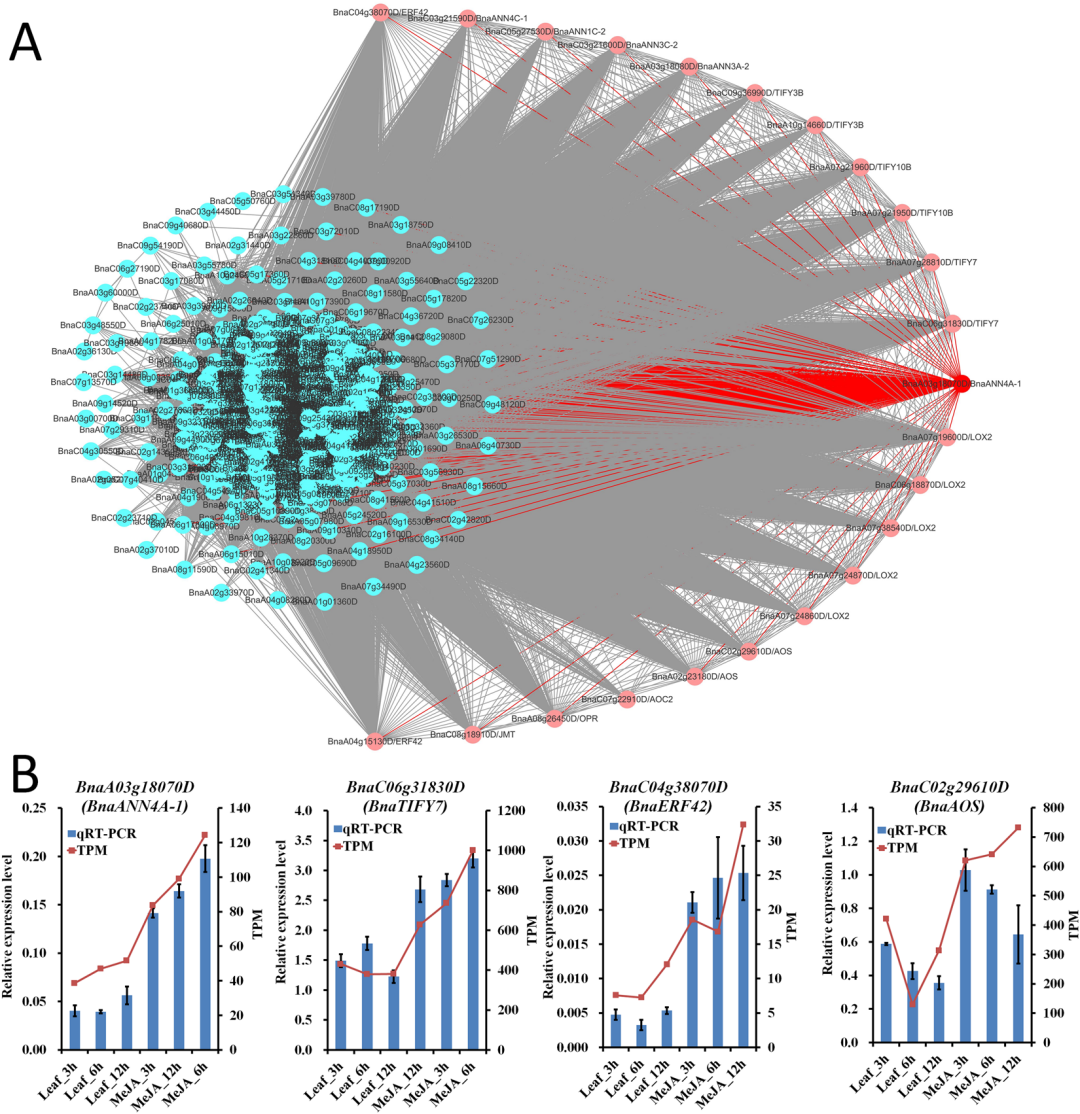
*BnaANN* involved in JA-response in *B. napus*. (**A**) Co-expression network for *BnaANN* genes in the lightgreen module. Red indicates candidate hub genes and light red indicates JA biosynthesis/responsive genes and *BnaANN* genes. (**B**) qRT-PCR analysis of four JA-response genes under MeJA treatments. The relative qRT-PCR expression level (blue bar) is shown on the left y-axis. The RNA-Seq TPM values (red line) are shown on the right y-axis. *BnaActin* (*BnaC05g34300D*) was used as the endogenous reference gene. The relative transcript levels were averaged over the three technical replicates.

Weighted gene co-expression network analysis (WGCNA) is an effective way to identify clusters of highly correlated genes^71^. To reveal the divergent functions of *BnaANN* genes in development, abiotic stress response, and hormone signaling, coexpression networks were constructed on the basis of pairwise correlations of all *B. napus* gene expression across 12 tissues samples and 8 treatment (abiotic stress and hormone) samples using WGCNA. The analysis identified 56 distinct modules (labeled with different colors) as shown in the dendrogram (Fig. S2). In total, 16 of 26 *BnaANN* genes were identified in six different modules: light green module (5), blue module (3), green module (3), turquoise module (3), salmon module (1), and magenta module (1) (Table S6). The lightgreen module (845 genes) was positively correlated with the MeJA treatment in leaves (Fig. S2). Five *BnaANN* genes (*BnaA03g18070D*, *BnaA03g18080D*, *BnaC03g21590D*, *BnaC03g21600D* and *BnaC05g27530D*) were induced by MeJA treatment in lightgreen module (Fig. 4 and Table S6). The top two hub genes with the highest the module membership kME (k-means clustering algorithm) values were *BnaA03g18070D* (*BnaANN*4A-1) and *BnaC06g31830D* (*BnaTIFY7*) in the light green module (Fig. 7A and Table S6). The jasmonate acid (JA) signaling repressor, TIFY, was induced by JA and regulates plant development and stress response^72–75^. Additionally, there were some *B. napus* JA biosynthesis genes and JA responsive genes in the light green module, such as the *Lipoxygenase* (*LOX*), *Allene oxide cyclase* (*AOC*), *Allene oxide synthase* (*AOS*), *12-oxophytodienoate reductase* (*OPR*), *Jasmonate O-methyltransferase* (*JMT*), and *Ethylene-responsive factor* (*ERF*) (Fig. 7A and Table S6). Transcriptional profiling and qRT-PCR analysis results showed that *BnaA03g18070D*/*BnaANN4A-1*, *BnaC06g31830D/BnaTIFY7, BnaC04g38070D/BnaERF42*, and *BnaC02g29610D/BnaAOS* were all induced by MeJA (Fig. 7B and Table S7). However, there was little research at the functions of annexins in JA signaling. *ZmAnx6.1* and *ZmAnx7* were induced at 12 h by JA, and the JA-responsive *cis*-elements exist in their promoters^76^. We analyzed the promotor sequences (2000 bp upstream of transcription start sites) of *BnaANN*, and founded that there were so many *cis*-elements involved in stress (drought, low-temperature, heat, anaerobic, wounding, defense and stress) response and plant hormones (MeJA, ABA and SA) response in their promotors, MeJA-responsive *cis*-element (CGTCA-motif, TGACG-motif and G-box) was the most numerous *cis*-element and all *BnaANN* members contain MeJA-responsive *cis*-elements (1 to 9) in promotors (Fig. S3). It suggested that the *BnaANN* genes in lightgreen module involved in JA signaling response in *B. napus*.

Three *BnaANN* genes (*BnaA04g22190D*, *BnaC03g49290D*, and *BnaC02g43450D*) in the blue module were expressed with NaCl and PEG treatments in roots, while genes (*BnaC08g06690D*, *BnaA10g20320D* and *BnaC09g44350D*) in the green module were expressed in roots. *BnaC01g16910D*, *BnaA06g23960D*, and *BnaC06g08410D* in the turquoise module were positively correlated with bud, stamen, ovule, and silique (Fig. S2 and Table S6). All the results indicate the different functions of *B. napus ANN* genes during plant development and stress response.

## Materials and Methods

### Identification of ANN of *B. rapa*, *B. oleracea*, and *B. napus*

*B. rape*, *B. oleracea* and *B. napus* ANN proteins have been identified using BLASTP (E-value < 1e-5) to look for homologs of *Arabidopsis* ANN among *B. rape*, *B. oleracea* and *B. napus* genome sequences database in *Ensembl gemones* (http://ensemblgenomes.org/)^77^. The annexin motifs in ANN proteins were characterized using InterPro (http://www.ebi.ac.uk/interpro/)^78^ and the NCBI conserved domain database (https://www.ncbi.nlm.nih.gov/Structure/cdd/wrpsb.cgi).

The molecular weight (Mw), isoelectric point (pI), and subcellular localization of ANN proteins were predicted using the Compute pI/Mw tool (http://web.expasy.org/compute_pi/)^79^ and ProtComp 9.0 (http://linux1.softberry.com/). The exon and intron organization of the *ANN* genes were analyzed using the Gene Structure Display Server (GSDS) (http://gsds.cbi.pku.edu.cn/)^80^. The conserved motifs of ANN were analyzed with MEME (http://meme.nbcr.net/meme/cgi-bin/meme.cgi)^81^.

### Phylogenetic analysis

Multiple sequence alignment of all identified ANN proteins (*Arabidopsis*, *B. rapa*, *B. oleracea*, and *B. napus*) were performed using ClustalW and a phylogenetic tree was constructed using the neighbour-joining (NJ) phylogenetic method in MEGA7^82^ with 1000 bootstrap replicates.

### Chromosomal localization of *ANN* genes

The position of *ANN* genes on the chromosomes of *B. rapa*, *B. oleracea*, and *B. napus* were obtained using TBtools v0.66831^83^.

### Nonsynonymous and sunonymous substitution rate ratio (*Ka/Ks*)

DnaSP (DNA Sequence Polymorphism) v6^84^ was used to calculate the ratio of the nonsynonymous substitution rate (*Ka*) to the synonymous substitution rate (*Ks*) and the *Ka/Ks* value between paralogous gene pairs.

### Plant materials and treatments

ZS11 (*B. napus* L. cv. Zhongshuang 11)^57^ seeds were allowed to germinate and then the seedlings were transplanted to pots containing soil or vermiculite. The growth conditions, hormone treatments, and abiotic stress conditions were as described previously^85^. Hormone treatments were performed by spraying leaves of 6-week-old seedlings with ABA (100 μM), MeJA (100 μM), SA (1 mM), and ETH (10 μg/ml); To simulate hot and cold stresses, seedlings were grown in chamber with 40 °C or 4 °C. To simulate salt and PEG stresses, seedlings were irrigated with NaCl (200 mM) or PEG-6000 (20%) solutions.

For chilling and freezing treatments with or without cold acclimation, the seedlings of two early-maturing semi-winter rapeseed varieties (HX17 and HX58) were used. They were treated as described previously^64^. Seedlings were cultured in incubators under 20 °C (14 h light: am6:00–pm8:00)/16 °C (10 h dark: pm8:00–am6:00) 4 weeks, then treated with 4 °C (14 days) → 4 °C (12 h) (CA) or −4 °C (12 h) (FA), 20 °C/16 °C (light/dark) 6 weeks → 4 °C (12 h) (CB), 20 °C (14 h light: am6:00–pm8:00)/16 °C (10 h dark: pm8:00–am6:00) 6 weeks → −4 °C (12 h) (FB). For the acclimation condition, after the 14 days at 4 °C, 4 °C/−4 °C (12 h) mean a treatment with 4 °C or −4 °C at pm8:00–am8:00 (10 h dark and 2 h light).

### RNA isolation and sequencing and gene expression analysis

The collected samples were sent to the sequencing cooperations of Sangon Biotech (Shanghai) Co., Ltd. and Novogene Co., Ltd. for RNA isolation, examination, and sequencing^64,85^. qRT-PCR analysis was performed as described previously^85^. The primers used in this study were listed in Table S8.

### Heat map analysis

The RPKM (Reads Per kb Per Million reads) and TPM (Transcripts Per Million) values were used to represent the expression levels of the *ANN* genes. A heat map of the expression profile of the *ANN* genes was plotted using Heatmap Illustrator, version 1.0^86^.

### Weighted gene coexpression network analysis (WGCNA)

Weighted gene coexpression network analysis was performed using WGCNA package in R^71^. The networks were visualized using Cytoscape v3^87^.

## Supplementary information

Supplementary information.

Supplementary information 2.

Supplementary information 3.

Supplementary information 4.

Supplementary information 5.

## Data Availability

The authors declare that all the data and plant materials will be available without restrictions.
