# Supplementary material for: Comprehensive analyses of the annexin (ANN) gene family in Brassica rapa, Brassica oleracea and Brassica napus reveals their roles in stress response
Source: Sci Rep. 2020 Mar 9;10:4295. doi: 10.1038/s41598-020-59953-w (PMC7062692; doi:10.1038/s41598-020-59953-w)
Supplement: Supplementary file 1 — Supplementary information. [file 41598_2020_59953_MOESM1_ESM.docx]

**Title:** Comprehensive analyses of the *annexin* (*ANN*) gene family in *Brassica* *rapa*, *Brassica* *oleracea* and *Brassica* *napus* reveals their roles in stress response

**Author list:** He Xin^1^, Liao Li^1^, Xie Sai^1^, Yao Min^1^, Xie Pan^1^, Liu Wei^1^, Kang Yu^1^, Huang Luyao^1^, Wang Mei^1^, Qian Lunwen^1^, Liu Zhongsong^2, 3^, Guan Chunyun^2, 3^, Guan Mei^2, 3^*, Hua Wei^1, 4,^ *

Figure S1: Sequence alignment of ANN proteins.

Multiple sequence alignment of all identified ANN proteins (*Arabidopsis*, *B. rapa*, *B. oleracea*, and *B. napus*) were performed using ClustalW.

**Figure S2:** Module-sample relationship detected based on gene coexpression networks in *B. napus*.

Each row represents a module and each column represents a tissue or a stress treatment. The color scale shows the correlation between each module and trait from −1 (blue) to 1 (red).

**Figure S3:** The *cis*-elements involved in stress-response and plant hormones response in *BnaANN* promotors (2000 bp upstream of transcription start sites).

Different colors indicate different cis-elements existing in the promoter region.
