# Supplementary material for: Comprehensive analyses of the annexin (ANN) gene family in Brassica rapa, Brassica oleracea and Brassica napus reveals their roles in stress response
Source: Sci Rep. 2020 Mar 9;10:4295. doi: 10.1038/s41598-020-59953-w (PMC7062692; doi:10.1038/s41598-020-59953-w)
Supplement: Supplementary file 2 — Supplementary information 2. [file 41598_2020_59953_MOESM2_ESM.pdf]

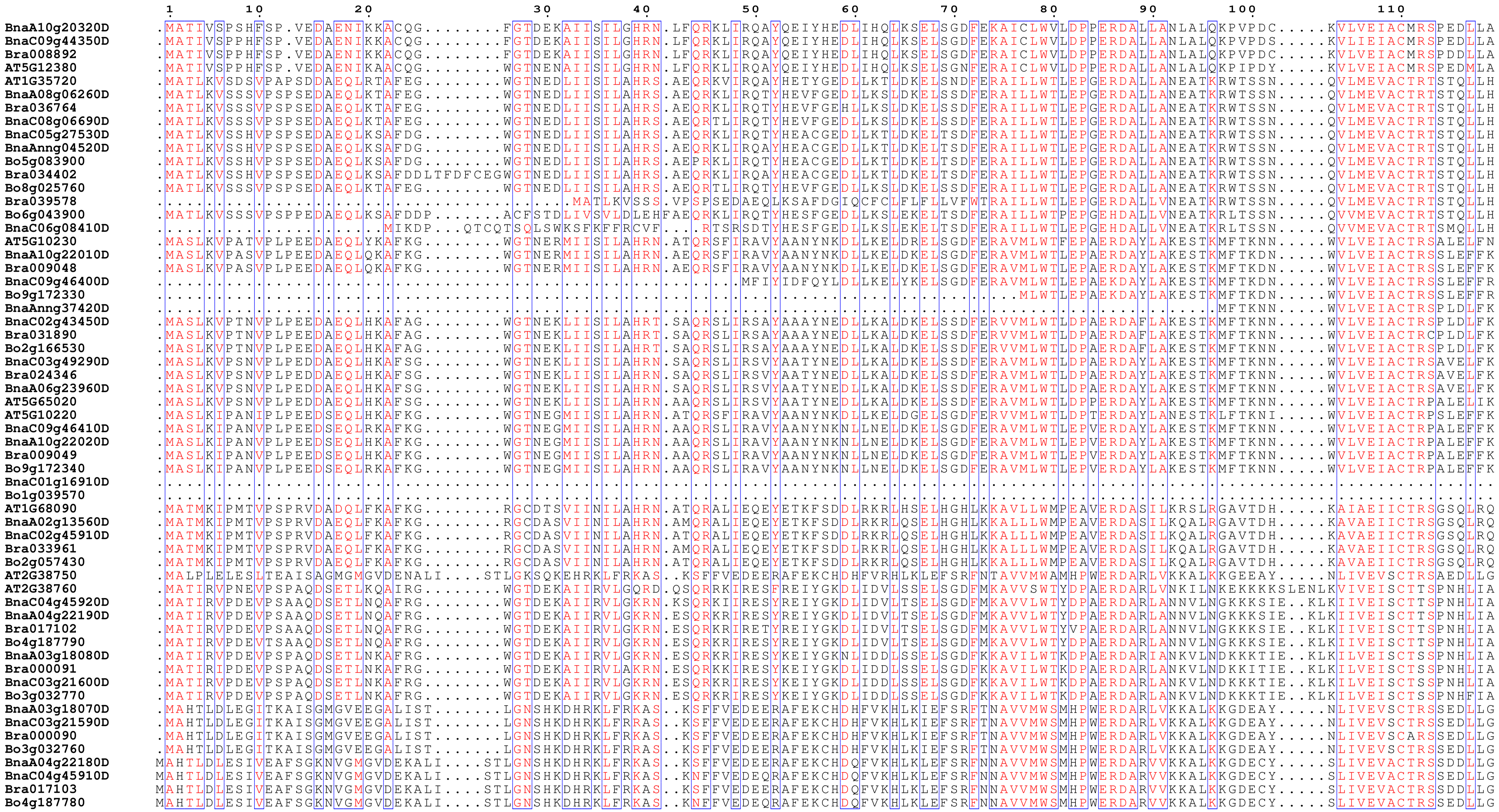
1 10 20 30 40 50 60 70 80 90 100 110
BnaA10g20320D .MATIVSPSHFSP.VEDAENIKKACQG........FGTDEKAIISILGHRN.LFQRKLIRQAYQEIYHEDLIHQLKSELSGDFEKAICLWVLDPPERDALLANLALQKPVPDC.....KVLVEIACMRSPEDLLA
BnaC09g44350D .MATIVSPPHFSP.VEDAENIKKACQG........FGTDEKAIISILGHRN.LFQRKLIRQAYQEIYHEDLIHQLKSELSGDFEKAICLWVLDPPERDALLANLALQKPVPDS.....KVLIEIACMRSPEDLLA
Bra008892 .MATIVSPPHFSP.VEDAENIKKACQG........FGTDEKAIISILGHRN.LFQRKLIRQAYQEIYHEDLIHQLKSELSGDFERAICLWVLDPPERDALLANLALQKPVPDC.....KVLVEIACMRSPDDLLA
AT5G12380 .MATIVSPPHFSP.VEDAENIKAACQG........WGTNENAIISILGHRN.LFQRKLIRQAYQEIYHEDLIHQLKSELSGNFERAICLWVLDPPERDALLANLALQKPIPDY.....KVLVEIACMRSPEDMLA
AT1G35720 .MATLKVSDSVPAPSDDAEQLRTAFEG........WGTNEDLIISILAHRS.AEQRKVIRQAYHETYGEDLLKTLDKELSNDFERAILLWTLEPGERDALLANEATKRWTSSN.....QVLMEVACTRTSTQLLH
BnaA08g06260D .MATLKVSSSVPSPSEDAEQLKTAFEG........WGTNEDLIISILAHRS.AEQRKLIRQTYHEVFGEDLLKSLDKELSSDFERAILLWTLEPGERDALLANEATKRWTSSN.....QVLMEVACTRTSTQLLH
Bra036764 .MATLKVSSSVPSPSEDAEQLKTAFEG........WGTNEDLIISILAHRS.AEQRKLIRQTYHEVFGEHLLKSLDKELSSDFERAILLWTLEPGERDALLANEATKRWTSSN.....QVLMEVACTRTSTQLLH
BnaC08g06690D .MATLKVSSSVPSPSEDAEQLKTAFEG........WGTNEDLIISILAHRS.AEQRTLIRQTYHEVFGEDLLKSLDKELSSDFERAILLWTLEPGERDALLANEATKRWTSSN.....QVLMEVACTRTSTQLLH
BnaC05g27530D .MATLKVSSHVPSPSEDAEQLKSAFDG........WGTNEDLIISILAHRS.AEQRKLIRQTYHEACGEDLLKTLDKELTSDFERAILLWTLEPGERDALLANEATKRWTSSN.....QVLMEVACTRTSTQLLH
BnaAnng04520D .MATLKVSSHVPSPSEDAEQLKSAFDG........WGTNEDLIISILAHRS.AEQRKLIRQTYHEACGEDLLKTLDKELTSDFERAILLWTLEPGERDALLANEATKRWTSSN.....QVLMEVACTRTSTQLLH
Bo5g083900 .MATLKVSSHVPSPSEDAEQLKSAFDG........WGTNEDLIISILAHRS.AEPRKLIRQTYHEACGEDLLKTLDKELTSDFERAILLWTLEPGERDALLANEATKRWTSSN.....QVLMEVACTRTSTQLLH
Bra034402 .MATLKVSSHVPSPSEDAEQLKSAFDDLTFDFCEGWGTNEDLIISILAHRS.AEQRKLIRQAYHEACGEDLLKTLDKELTSDFERAILLWTLEPGERDALLANEATKRWTSSN.....QVLMEVACTRTSTQLLH
Bo8g025760 .MATLKVSSSVPSPSEDAEQLKTAFEG........WGTNEDLIISILAHRS.AEQRTLIRQTYHEVFGEDLLKSLDKELSSDFERAILLWTLEPGERDALLANEATKRWTSSN.....QVLMEVACTRTSTQLLH
Bra039578 ....MATLKVSSS.VPSPSEDAEQLKSAFDGIQCFCLFLFLLVFWTRAILLWTLEPGERDALLVNEATKRWTSSN.....QVLMEVACTRTSTQLLH
Bo6g043900 .MATLKVSSSVPSPPEDAEQLKSAFDDP.......ACFSTDLIVSVLDLEHFAEQRKLIRQTYHESFGEDLLKSLEKELTSDFERAILLWTLEPGEHDALLVNEATKRLTSSN.....QVVMEVACTRTSTQLLH
BnaC06g08410D .......................MIKDP...QTCQTSQLSWKSFKFFRCVF....RTSRSDTYHESFGEDLLKSLEKELTSDFERAILLWTLEPGEHDALLVNEATKRLTSSN.....QVVMEVACTRTSMQLLH
AT5G10230 .MASLKVPATVPLPEEDAEQLYKAFKG........WGTNERMIISILAHRN.ATQRSFIRAVYAANYNKDLLKELDRELSGDFERAVMLWTFEPAERDAYLAKESTKMFTKNN.....WVLVEIACTRSALELFN
BnaA10g22010D .MASLKVPASVPLPEEDAEQLQKAFKG........WGTNERMIISILAHRN.AEQRSFIRAVYAANYNKDLLKELDKELSGDFERAVMLWTLEPAERDAYLAKESTKMFTKDN.....WVLVEIACTRSSLEFFK
Bra009048 .MASLKVPASVPLPEEDAEQLQKAFKG........WGTNERMIISILAHRN.AEQRSFIRAVYAANYNKDLLKELDKELSGDFERAVMLWTLEPAERDAYLAKESTKMFTKDN.....WVLVEIACTRSSLEFFK
BnaC09g46400D ....MFIYIDFQYLDLLKELYKELSGDFERAVMLWTLEPAEKDAYLAKESTKMFTKDN.....WVLVEIACTRSSLEFFR
Bo9g172330 ....MLWTLEPAEKDAYLAKESTKMFTKDN.....WVLVEIACTRSSLEFFR
BnaAnng37420D ....MFTKNN.....WVLVEIACTRSPLDLFK
BnaC02g43450D .MASLKVPTNVPLPEEDAEQLHKAFAG........WGTNEKLIISILAHRT.SAQRSLIRSAYAAAYNEDLLKALDKELSSDFERVVMLWTLDPAERDAFLAKESTKMFTKNN.....WVLVEIACTRSPLDLFK
Bra031890 .MASLKVPTNVPLPEEDAEQLHKAFAG........WGTNEKLIISILAHRT.SAQRSLIRSAYAAAYNEDLLKALDKELSSDFERVVMLWTLDPAERDAFLAKESTKMFTKNN.....WVLVEIACTRCPLDLFK
Bo2g166530 .MASLKVPTNVPLPEEDAEQLHKAFAG........WGTNEKLIISILAHRT.SAQRSLIRSAYAAAYNEDLLKALDKELSSDFERVVMLWTLDPAERDAFLAKESTKMFTKNN.....WVLVEIACTRSPLDLFK
BnaC03g49290D .MASLKVPSNVPLPEDDAEQLHKAFSG........WGTNEKLIISILAHRN.SAQRSLIRSVYAATYNEDLLKALDKELSSDFERAVMLWTLDPAERDAYLAKESTKMFTKNN.....WVLVEIACTRSAVELFK
Bra024346 .MASLKVPSNVPLPEDDAEQLHKAFSG........WGTNEKLIISILAHRN.SAQRSLIRSVYAATYNEDLLKALDKELSSDFERAVMLWTLDPAERDAYLAKESTKMFTKNN.....WVLVEIACTRSAVELFK
BnaA06g23960D .MASLKVPSNVPLPEDDAEQLHKAFSG........WGTNEKLIISILAHRN.SAQRSLIRSVYAATYNEDLLKALDKELSSDFERAVMLWTLDPAERDAYLAKESTKMFTKNN.....WVLVEIACTRSAVELFK
AT5G65020 .MASLKVPSNVPLPEDDAEQLHKAFKG........WGTNEKLIISILAHRN.AAQRSLIRSVYAATYNEDLLKALDKELSSDFERAVMLWTLDPPERDAYLAKESTKMFTKNN.....WVLVEIACTRPALELIK
AT5G10220 .MASLKIPANIPLPEEDSEQLHKAFKG........WGTNEGMIISILAHRN.ATQRSFIRAVYAANYNKDLLKELDGELSGDFERVVMLWTLDPTERDAYLANESTKLFTKNI.....WVLVEIACTRPSLEFFK
BnaC09g46410D .MASLKIPANVPLPEEDSEQLRKAFKG........WGTNEGMIISILAHRN.AAQRSLIRAVYAANYNKNLLNELDKELSGDFERAVMLWTLEPVERDAYLAKESTKMFTKNN.....WVLVEIACTRPALEFFK
BnaA10g22020D .MASLKIPANVPLPEEDSEQLHKAFKG........WGTNEGMIISILAHRN.AAQRSLIRAVYAANYNKNLLNELDKELSGDFERAVMLWTLEPVERDAYLAKESTKMFTKNN.....WVLVEIACTRPALEFFK
Bra009049 .MASLKIPANVPLPEEDSEQLHKAFKG........WGTNEGMIISILAHRN.AAQRSLIRAVYAANYNKNLLNELDKELSGDFERAVMLWTLEPVERDAYLAKESTKMFTKNN.....WVLVEIACTRPALEFFK
Bo9g172340 .MASLKIPANVPLPEEDSEQLRKAFKG........WGTNEGMIISILAHRN.AAQRSLIRAVYAANYNKNLLNELDKELSGDFERAVMLWTLEPVERDAYLAKESTKMFTKNN.....WVLVEIACTRPALEFFK
BnaC01g16910D ....
Bo1g039570 ....
AT1G68090 .MATMKIPMTVPSPRVDADQLFKAFKG........RGCDTSVIINILAHRN.ATQRALIEQEYETKFSDDLRKRLHSELHGHLKKAVLLWMPEAVERDASILKRSLRGAVTDH.....KAIAEIICTRSGSQLRQ
BnaA02g13560D .MATMKIPMTVPSPRVDAEQLFKAFKG........RGCDASVIINILAHRN.AMQRALIEQEYETKFSDDLRKRLQSELHGHLKKALLLWMPEAVERDASILKQALRGAVTDH.....KAVAEIICTRSGSQLRQ
BnaC02g45910D .MATMKIPMTVPSPRVDAEQLFKAFKG........RGCDASVIINILAHRN.ATQRALIEQEYETKFSDDLRKRLQSELHGHLKKALLLWMPEAVERDASILKQALRGAVTDH.....KAVAEIICTRSGSQLRQ
Bra033961 .MATMKIPMTVPSPRVDAEQLFKAFKG........RGCDASVIINILAHRN.AMQRALIEQEYETKFSDDLRKRLQSELHGHLKKALLLWMPEAVERDASILKQALRGAVTDH.....KAVAEIICTRSGSQLRQ
Bo2g057430 .MATMKIPMTVPSPRVDAEQLFKAFKG........RGCDASVIINILAHRN.ATQRALIEQEYETKFSDDLRKRLQSELHGHLKKALLLWMPEAVERDASILKQALRGAVTDH.....KAVAEIICTRSGSQLRQ
AT2G38750 .MALPLELESLTEAISAGMGMGVDENALI....STLGKSQKEHRKLFRKAS..KSFFVEDEERAFEKCHDHFVRHLKLEFSRFNTAVVMWAMHPWERDARLVKKALKKGEEAY.....NLIVEVSCTRSAEDLLG
AT2G38760 .MATIRVPNEVPSPAQDSETLKQAIRG........WGTDEKAIIRVLGQRD.QSQRRKIRESFREIYGKDLIDVLSSELSGDFMKAVVSWTYDPAERDARLVNKILNKEKKKSLENLKVIVEISCTTSPNHLIA
BnaC04g45920D .MATIRVPDEVPSAAQDSETLNQAFRG........WGTDEKAIIRVLGKRN.KSQRKIIRESYREIYGKDLIDVLTSELSGDFMKAVVLWTYDPAERDARLANNVLNGKKKSIE..KLKIIVEISCTTSPNHLIA
BnaA04g22190D .MATIRVPDEVPSAAQDSETLNQAFRG........WGTDEKAIIRVLGKRN.ESQRKRIRESYREIYGKDLIDVLTSELSGDFMKAVVLWTYDPAERDARLANNVLNGKKKSIE..KLKIIVEISCTTSPNHLIA
Bra017102 .MATIRVPDEVPSAAQDSETLNQAFRG........WGTDEKAIIRVLGKRN.ESQRKRIRETYREIYGKDLIDVLTSELSGDFMKAVVLWTYVPAERDARLANNVLNGKKKSIE..KLKIIVEISCTTSPNHLIA
Bo4g187790 .MATIRVPDEVTSAAQDSETLNQAFRG........WGTDEKAIIRVLGKRN.ESQRKRIRESYREIYGKDLIDVLTSELSGDFMKAVVLWTYDPAERDARLANNVLNGKKKSIE..KLKIIVEISCTTSPNHLIA
BnaA03g18080D .MATIRVPDEVPSPAQDSETLNKAFRG........WGTDEKAIIRVLGKRN.ESQRKRIRESYKEIYGKNLIDDLSSELSGDFKKAVILWTKDPAERDARIANKVLNDKKITIE..KLKILVEISCTSSPNHLIA
Bra000091 .MATIRIPDEVPSPAQDSETLNKAFRG........WGTDEKAIIRVLAKRN.ESQRKKIRESYKEIYGKDLIDDLSSELSGDFKKAVILWTKDPAERDARIANKVLNDKKITIE..KLKILVEISCTSSPNHLIA
BnaC03g21600D .MATIRVPDEVPSPAQDSETLNKAFRG........WGTDEKAIIRVLGKRN.ESQRKRIRESYKEIYGKDLIDDLSSELSGDFKKAVILWTKDPAERDARLANKVLNDKKKTIE..KLKILVEISCTSSPNHLIA
Bo3g032770 .MATIRVPDEVPSPAQDSETLNKAFRG........WGTDEKAIIRVLGKRN.ESQRKRIRESYKEIYGKDLIDDLSSELSGDFKKAVILWTKDPAERDARLANKVLNDKKKTIE..KLKILVEISCTSSPNHFIA
BnaA03g18070D .MAHTLDLEGITKAISGMGVEEGALIST.......LGNSHKDHRKLFRKAS..KSFFVEDEERAFEKCHDHFVKHLKIEFSRFTNAVVMWSMHPWERDARLVKKALKKGDEAY.....NLIVEVSCTRSSEDLLG
BnaC03g21590D .MAHTLDLEGITKAISGMGVEEGALIST.......LGNSHKDHRKLFRRAS..KSFFVEDEERAFEKCHDHFVKHLKIEFSRFTNAVVMWSMHPWERDARLVKKALKKGDEAY.....NLIVEVSCTRSSEDLLG
Bra000090 .MAHTLDLEGITKAISGMGVEEGALIST.......LGNSHKDHRKLFRKAS..KSFFVEDEERAFEKCHDHFVKHLKIEFSRFTNAVVMWSMHPWERDARLVKKALKKGDEAY.....NLIVEVSCARSSEDLLG
Bo3g032760 .MAHTLDLEGITKAISGMGVEEGALIST.......LGNSHKDHRKLFRRAS..KSFFVEDEERAFEKCHDHFVKHLKIEFSRFTNAVVMWSMHPWERDARLVKKALKKGDEAY.....NLIVEVSCTRSSEDLLG
BnaA04g22180D MAHTLDLESIVEAFSGKNVGMGVDEKALI....STLGNSHKDHRKLFRKAS..KSFFVEDEERAFEKCHDQFVKHLKLEFSRFNNAVVMWSMHPWERDARVVKKALKKGDECY.....SLIVEVACTRSSDDLLG
BnaC04g45910D MAHTLDLESIVEAFSGKNVGMGVDEKALI....STLGNSHKDHRKLFRKAS..KNFFVEDEQRAFEKCHDQFVKHLKLEFSRFNNAVVMWSMHPWERDARVVKKALKKGDECY.....SLIVEVACTRSSEDLLG
Bra017103 MAHTLDLESIVEAFSGKNVGMGVDEKALI....STLGNSHKDHRKLFRKAS..KSFFVEDEERAFEKCHDQFVKHLKLEFSRFNNAVVMWSMHPWERDARVVKKALKKGDECY.....SLIVEVACTRSSDDLLG
Bo4g187780 MAHTLDLESIVEAFSGKNVGMGVDEKALI....STLGNSHKDHRKLFRKAS..KNFFVEDEQRAFEKCHDQFVKHLKLEFSRFNNAVVMWSMHPWERDARVVKKALKKGDECY.....SLIVEVACTRSEDLLG

```
                   120       130       140       150       160       170       180       190       200       210       220       230       240
BnaA10g20320D      TRRAYRCLYKRSLEEDLASRTTGDIRKLLVATVSAYKYDGDEI..DETLAHSEAAI...LHDEILDKAVGHEETIRVISTRTSVQLCAIFNRYKDVYGRSITKDILSHPTNEYLSALRAAIR.CTKNPNRYYAKV
BnaC09g44350D      TRRAYRCLYKRSLEEDLASRTTGDIRKLLVATASAYKYDGEEF..DETLAHSEAAI...LHDEILGKAVDHEETIRVISTRSGVQLCAIFNRYKDIYGRSITKDMLSHPTNEYLSALRAAIR.CIKNPNRYYAKV
Bra008892          TRRAYRCLYKRSLEEDLASRTTGDIRKLLVATASAYKYDGEEI..DETLAHSEAAI...LHDEILGKAVDHEETIRVISTRSSMQLCAIFNRYKDLYGRSITKDILSHPTNEYLSALRAAIR.CIKNPNRYYAKV
AT5G12380          ARRAYRCLYKHSLEEDLASRTIGDIRRLLVAMVSAYKYDGEEI..DEMLAQSEAAI...LHDEILGKAVDHEETIRVLSTRSSMQLSAIFNRYKDIYGTSITKDLLNHPTNEYLSALRAAIR.CIKNPTRYYAKV
AT1G35720          ARQAYHARYKKSLEEDVAHHTTGDFRKLLVSLVTSYRYEGDEV..NMTLAKQEAKL...VHEKIKDKHYNDEDVIRILSTRSKAQINATFNRYQDDHGEEILKSLEEGDDDDKFLALLRSTIQCLTRPELYFVDV
BnaA08g06260D      ARQAYHARFKKSIEEDVAHHTTGDFRKLLVSLVSSYRYEGDEV..NMTLAKQEAKL...IHERIKDKHYSDEEVIRILSTRSKAQINATFNRYQDDHGEEILKSLEEGDGDDKFLELLRSTIQCLTRPELYFVDV
Bra036764          ARQAYHARFKKSIEEDVAHHTTGDFRKLLVSLVSSYRYEGDEV..NMTLAKQEAKL...IHERIKDKHYSDEEVIRILSTRSKAQINATFNRYQDDHGEEILKSLEEGDGDDKFLELLRSTIQCLTRPELYFVDV
BnaC08g06690D      ARQAYHARFKKSIEEDVAHHTTGDFRKLLVSLVSSYRYEGDEV..NMTLAKQEAKL...IHERIKDKHYSDEEVIRILSTRSKAQINATFNRYQDDHGEEILKSLEEGYGDDKFLELLRSTIQCLTRPELYFVDV
BnaC05g27530D      ARQAYHARYKKSIEEDVAHHTTGDFRKLLVSLVSSYRYEGDEV..NMTLATQEAKL...IHEKIKDKHYSDEDVIRILSTRSKAQINATFNRYQDEHGEEILKSLEEGDEDDKFLGLLRSTIQCLTRPELYFVDV
BnaAnng04520D      ARQAYHARYKKSIEEDVAHHTTGDFRKLLVSLVSSYRYEGDEV..NMTLAKQEAKL...IHEKIKDKHYSDEDVIRILSTRSKAQINATFNRYQDEHGEEILKSLEEGDEDDKFLGLLRSTIQCLTRPELYFVDV
Bo5g083900         ARQAYHARYKKSIEEDVAHHTTGDFRKLLVSLVSSYRYEGDEV..NMTLAKQEAKL...IHEKIKDKHYSDEDVIRILSTRSKAQINATFNRYQDEHGEEILKSLEEGDEDDKFLGLLRSTIQCLTRPELYFVDV
Bra034402          ARQAYHARYKKSIEEDVAHHTTGDFRKLLVSLVSSYRYEGDEV..NMTLAKQEAKL...IHEKIKDKHYSDEDVIRILSTRSKAQINATFNRYQDEHGEEILKSLEEGDEDDKFLGLLRSTIQCLTRPELYFVDV
Bo8g025760         ARQAYHARFKKSIEEDVAHHTTGDFRKLLVSLVSSYRYEGDEV..NMTLAKQEAKL...IHERIKDKHYSDEEVIRILSTRSKAQINATFNRYQDDHGEEILKSLEEGYGDDKFLELLRSTIQCLTRPELYFVDV
Bra039578          ARQAYHARYKKSIEEDVAHHTTGDFRKLLVSLVSSYRYEGEEV..NMTLAKQEAKL...IHEKIKDKHYNDEDFIRILSTRSKPQINATFNRYQDNHGEEILKSLEEGDEDDKFLGLLRSTIQCLTRPELYFVDV
Bo6g043900         ARQAYHARFKKSIEEDVAHHTTGDFKKLLVSLVSSYRYEGEEV..NMTLAKQEAKL...IHEKIKDKHYNDEDIIRILSTRRKSQINATFNRYQDNHGEEILKSLEEGDEDDKFLGLLRSTIQCLTRPELYFVDV
BnaC06g08410D      ARQAYHARFKKSIEEDVAHHTTGDFRKLLVSLVSSYRYEGEEV..NMTLAKQEAKL...IHEKIKDKHYNDEDIIRILSTRRKAQINATFNRYQDNHGEEILKSLEEGDDDDKFLGLLRSTIQ............
AT5G10230          AKQAYQARYKTSLEEDVAYHTSGDIRKLLVPLVSTFRYDGDEV..NMTLARSEAKI...LHEKIKEKAYADDDLIRILTTRSKAQISATLNHYKNNFGTSMSKYLKEDSENEYIQLLKAVIK.CLTYPEKYFEKV
BnaA10g22010D      AKQAYQVRYKTSIEEDVAYHTSGDVRKLLVPLVSTFRYDGDEV..NMMIAKSEAKI...LHEKMEAKDYNDGDLIRILTTRSKAQISATLNHFKNKFGTSITKYLKEDSDNEYVQLLKAVIK.CLTYPEKYFEKV
Bra009048          AKQAYQVRYKTSIEEDVAYHTSGDVRKLLVPLVSTFRYDGDEV..NMMIAKSEAKI...LHEKMEAKDYNDGDLIRILTTRSKAQISATLNHFKNKFGTSITKYLKEDSDNEYVQLLKAVIK.CLTYPEKYFEKV
BnaC09g46400D      AKQAYQVGYKTSIEEDVAYHTSGGVRKLLVPLVSTFRYDGDEV..NMMIAKSEAKI...LHEKMEAKDYNDGDLIRILTTRSKAQISATLNHFKNKFGTSITKYLKEDSDNEYVQLLKAVIK.CLTYPEKYFEKV
Bo9g172330         AKQAYQVGYKTSIEEDVAYHTSGGVRKLLVPLVSTFRYDGDEV..NMMIAKSEAKI...LHEKMEAKDYNDGDLIRILTTRSKAQISATLNHFKNKFGTSITKYLKEDSDNEYVQLLKAVIK.CLTYPEKYFEKV
BnaAnng37420D      VKQAYQARYKKSLEEDVAQHTSGDLRRLLLPLVSTFRYEGDEV..NMRLARSEAKL...LHEKVSEKAFSDDDFIRILTTRSKAQLGATLNHYNNEYGNAINKHLKEDSDDEYLKLLRAAIT.CLTYPEKHFEKV
BnaC02g43450D      VKQAYQARYKKSLEEDVAQHTSGDLRKLLLPLVSTFRYEGDEV..NMRLARSEAKL...LHEKVSEKAFSDDDFIRILTTRSKAQLGATLNHYNNEYGNAINKHLKEDSDDEYLKLLRAAIT.CLTYPEKHFEKV
Bra031890          VKQAYQARYKKSLEEDVAQHTSGDLRKLLLPLVSTFRYEGDEV..NMRLARSEAKL...LHEKVSEKAFSDDDFIRILTTRSKAQLGATLNHYNNEYGNAINKHLKEDSDDEYLKLLRAAIT.CLTYPEKHFEKV
Bo2g166530         VKQAYQARYKKSLEEDVAQHTSGDLRKLLLPLVSTFRYEGDEV..NMRLARSEAKL...LHEKVSEKAFSDDDFIRILTTRSKAQLGATLNHYNNEYGNAINKHLKEDSDDEYLKLLRAAIT.CLTYPEKHFEKV
BnaC03g49290D      VKQAYQARYKKSLEEDVAQHTSGDLRKLLLPLVSTFRYEGDDV..NMMLARSEAKL...LHEKVSEKAFNDDDFIRILTTRSKAQLGATLNHYNNEHGNSINKNLKEGSDDDYLKLLRAAIT.CLTYPEKHFEKV
Bra024346          VKQAYQARYKKSLEEDVAQHTSGDLRKLLLPLVSTFRYEGDDV..NMMLARSEAKL...LHEKVSEKAFNDDDFIRILTTRSKAQLGATLNHYNNEHGNSINKNLKEGSDDDYLKLLRAAIT.CLTYPEKHFEKV
BnaA06g23960D      VKQAYQARYKKSLEEDVAQHTSGDLRKLLLPLVSTFRYEGDDV..NMMLARSEAKL...LHEKVSEKAFNDDDFIRILTTRSKAQLGATLNHYNNEHGNSINKVTTSTTLQCCPVFWFGSVR............
AT5G65020          VKQAYQARYKKSIEEDVAQHTSGDLRKLLLPLVSTFRYEGDDV..NMMLARSEAKI...LHEKVSEKSYSDDDFIRILTTRSKAQLGATLNHYNNEYGNAINKNLKEESDDNDYMKLLRAVIT.CLTYPEKHFEKV
AT5G10220          TKQAYHVRYKTSLEEDVAYHTSGNIRKLLVPLVSTFRYDGNADEVNVKLARSEAKT...LHKKITEKAYTDEDLIRILTTRSKAQIMATLNHFKDKFGSSINKFLKEDSNDDYVQLLKTAIK.CLTYPEKYFEKV
BnaC09g46410D      AKQAYQARYKTSLEEDVAYHTSGDIRKLLVPLVSTFRYDGNADEVNMKLASSEAKT...LHKKITEKAYTDEDLIRILTTRSKAQIMATLNHFKDKFGSSINKYLKEDSEDEYVQLLKTTIK.CLTYPEKYYEKV
BnaA10g22020D      AKQAYQAHYKTSLEEDVAYHTSGDIRKLLVPLVSTFRYDGNADEVNMKLASSEAKT...LHKKITEKAYTDEDLIRILTTRSKAQIMATLNHFKDKFGSSINKYLKEDSEDEYVQLLKTAIK.CLTYPEKYNEKV
Bra009049          AKQAYQAHYKTSLEEDVAYHTSGDIRKLLVPLVSTFRYDGNADEVNMKLASSEAKT...LHKKITEKAYTDEDLIRILTTRSKAQIMATLNHFKDKFGSSINKYLKEDSEDEYVQLLKTAIK.CLTYPEKYNEKV
Bo9g172340         AKQAYQARYKTSLEEDVAYHTSGDIRKLLVPLVSTFRYDGNADEVNMKLASSEAKT...LHKKITEKAYTDEDLIRILTTRSKAQIMATLNHFKDKFGSSINKYLKEDSEDEYVQLLKTTIK.CLTYPEKYYEKV
BnaC01g16910D      ..........................................MALRAVG..VYMLLLDLREK...GMRIFLKKVWSVVELCRTEVTNYDLQGS.TRVHHHNPNSKPLEGLPFTVLTAQTC...NHNMPLTEQHKEERENSV
Bo1g039570         ..........................................MALRAVG..VYMLLLDLREK...GMRIFLKKVWSVVELCRTEVTNYDLQGS.TRVHHHNPNSKPLEGLPFTVLTAQTC...NHNMPLTEQHKEERENSV
AT1G68090          IKQVYSNTFGVKLEEDIESEASGNHKRVLLAYLNTTRYEGPEI.DNASVENDARTL...KSAVARKHKSDDQTLIQIFTDRSRTHLVAVRSTYRSMYGKELGKAIR.DETRGNFEHVLLTILQCAENSCFYFAKA
BnaA02g13560D      IKQVYLNTFGVRVEQDVESEASGNHKRVLLAYLNTTRYEGPEI..DDKIVENDARV...LKKAVARKHKSDDQTLIQIFTDRSRTHLVALRSTYRSMFGKELGKAIRDETRGNFEHVLLTILQCAENSSFYFAKA
BnaC02g45910D      IKQVYLNTFGVRVEQDVESEASGNHKRVLLAYLNTTRYEGPEI..DDKIVENDARV...LKKAVARKHKSDDQTLIQIFTDRSRTHLVALRSTYRSMFGKELGKAIRDETRGNFEHVLLTILQCAENSSFYFAKA
Bra033961          IKQVYLNTFGVRVEQDVESEASGNHKRVLLAYLNTTRYEGPEI..DDKIVENDARV...LKKAVARKHKSDDQTLIQIFTDRSRTHLVALRSTYRSMFGKELGKAIRDETRGNFEHVLLTILQCAENSSFYFAKA
Bo2g057430         IKQVYLNTFGVRVEQDVESEASGNHKRVLLAYLNTTRYEGPEI..DDKIVENDARV...LKKAVARKHKSDDQTLIQIFTDRSRTHLVALRSTYRSMFGKELGKAIRDETRGNFEHVLLTILQCAENSSFYFAKA
AT2G38750          ARKAYHSLFDQSMEEDIASHVHGPQRKLLVGLVSAYRYEGNKV..KDDSAKSDAKILAEAVASSGEEAVEKDEVVRILTTRSKLHLQHLYKHFNEIKGSDLLGGVSKSSLLNEALICLLKPALYFSKILDASLNK
AT2G38760          VRKAYCSLFDSSLEEHIASSLPFPLAKLLVTLASTFRYDKDRT..DAEVATIEAAM...LREAIEKKQLDHDHVLYILGTRSIYQLRETFVAYKKNYGVTIDKDVDGCPGDADLRSLLKVAIFCIDTPEKHFAKV
BnaC04g45920D      VRKAYCSLFDLSLEEHIASSVPFPLAKVLVTLATSFRYDKDMA..DTEVATIEAGM...LREAITAKKLDHDHVLYILGTRSIYQLRATFVAYKQSYENTLDKDVDGCAGDADLKSLLQMVILCIEFPEKHFAKV
BnaA04g22190D      VRKAYCSLFDLSLEEHIASSVPFPLAKVLVTLATSFRYDKDMA..DTEVATIEAGM...LREAITAKQLDHDHVLYILGTRSIYQLRETFVAYKQSYGSTLDKDVDGCPGDADLKSLLQVVILCIEFPEKHFAKV
Bra017102          VRKAYCSLFDLSLEEHIASSVPFPLAKVLVTLATSFRYDKDMA..DTEVATIEAGM...LREAITAKQLDHDHVLYILGTRSIYQLRETFVAYKQSYGNTLDKDVDGCPGDADLKSLLQMVILCIEFPEKHFAKV
Bo4g187790         VRKAYCSLFDLSLEEHIASSVPFPLAKVLVTLATSFRYDKDMA..DTEVATIEAGM...LREAITAKKLDHDHVLYILGTRSIYQLRATFVAYKQSYENTLDKDVDGCPGDADLKSLLQMVILCIEFPEKHFAKV
BnaA03g18080D      VRKAYCSLYDSSLEEDIASSVPFPLAKLLVTLATSFRYDKEKV..DKEVATIEAGM...LHEAISKKQLDHDHVLYIIGTRSIYQLRATFVAYKQSYGNTLDKDVDGCPGEADLRSLLQMVILCIEFPEKHFAKV
Bra000091          VRKAYCSLYDSSLEQDIASSVPFPLAKLLVTLATSFRYDKEKV..DKEVATIEAGM...LHEAISKKQLDHDHVLYIIGTRSIYQLRATFVAYKQSYGNTLDKDVDGCPGDADLRSLLQMVILCIEFPEKHFAKV
BnaC03g21600D      VRKAYCSLYDSSLEQDIASSVPFPLAKLLVTLATSFRYDKEKV..DKEVATIEAGM...LHEAISKKQLDHDHVLYIIGTRSIYQLRETFVAYKQSYGNTLDKDVDGCPGDADLRSLLQKVILCIEFPEKHFAKV
Bo3g032770         VRKAYCSLYDSSLEEDIASSVPFPLAKLLVTLATSFRYDKEKV..DKEVATIEAGM...LHEAISKKQLDHDHVLYIIGTRSIYQLRETFVAYKQSYGNTLDKDVDGCPGDADLRSLLQMVILCIEFPEKHFAKV
BnaA03g18070D      ARKAYHSLFDQSMEEDIASHVHGSQRKLLVGLVSAYRYEGKEV..KESSAKSDAKILAEAVASSGEGAIENDEVVRILSTRSKLHLEHLYKHFNQIKGSDLLGGVSESSLINEALLCLLKPS....VYFSKILNA
BnaC03g21590D      ARKAYHSLFDQSMEEDIASHVHGSQRKLLVGLVSAYRYEGKEV..KESSAKSDAKILAEAVASSGEGAIENDEVVRILSTRSKLHLEHLYKHFNQIQGSDLLGGVSESSLINEALLCLLKPS....VYFSKILNA
Bra000090          ARKAYHSLFDQSMEEDIASHVHGSQRKLLVGLVSAYRYEGKEV..RESSAKSDAKILAEAVASSGEGAIENDEVVRILSTRSKLHLEHLYKHFNQIKGSDLLGGVSESSLINEALLCLLKPS....VYFSKILNA
Bo3g032760         ARKAYHSLFDQSMEEDIASHVHGSQRKLLVGLVSAYRYEGKEV..KESSAKSDAKILAEAVASSGEGAIENDEVVRILSTRSKLHLEHLYKHFNQIQGSDLLGGVSESSLINEALLCLLKPS....VYFSKILNA
BnaA04g22180D      ARKAYHSLFDQSMEEDIASNIHGSRRKLLVGLVSAYRYEGNKV..NDDLAKSDAKILAEAVASPGEKAIENDEVVRILSTRSKLHIQQLYKHFNQINGSDLLGGVSDSSILNDALLCLLKPSVYFSKILNASLNK
BnaC04g45910D      ARKAYHSLFDQSMEEDIASNIHGSRRKLLVGLVSAYRYEGNKV..KDDSAKSDAKILAEAVASPGEKAIENDEVVRILSTRSKLHLQQLYKHFNQIKGSDLLVGVSDSSLLNDALLCLLKPSVYFSKILNASLNK
Bra017103          ARKAYHSLFDQSMEEDIASNIHGSRRKLLVGLVSAYRYEGNKV..NDDLAKSDAKILAEAVASPGEKAIENDEVVRILSTRSKLHLQQLYKHFNQINGSDLLGGVSDSSILNDALLCLLKPSVYFSKILNASLNK
Bo4g187780         ARKAYHSLFDQSMEEDIASNIHGSRRKLLVGLVSAYRYEGNKV..KDDSAKSDAKILAEAVASPGEKAIENDEVVRILSTRSKLHLQQLYKHFNQIKGSDLLAGVSDSSLLNDALLCLLKPSVYFSKILNASLNK
```

```
                 250       260       270       280       290       300       310
BnaA10g20320D   LRNAINTAGTDEDALTRVIVTRAEKDLKNITELYHKRNNESLDQAIAKETSRDYKAFLLALLGHGGI.
BnaC09g44350D   LRNAINTAGTDEDALTRVIVTRAEKDLKKITELYHKRNNESLDQAIAKETSRDYKAFLLALLGHGGI.
Bra008892       LRNAINTAGTDEDALTRVIVTRAEKDLKNITELYHKRNNESLDQAIAKETSRDYKAFLLALLGHGGI.
AT5G12380       LRNSINTVGTDEDALNRVIVTRAEKDLTNITGLYFKRNNVSLDQAIAKETSGDYKAFLLALLGHGKQL
AT1G35720       LRSAINKTGTDEGALTRIVTTRAEIDLKVIGEEYQRRNSIPLEKAITKDTRGDYEKMLVALLGEDDA.
BnaA08g06260D   LRSAINKTGTDEGALTRIVTTRAEIDLKVIGEEYQRRNSIPLEKAITKDTRGDYEKMLVALLGEDDA.
Bra036764       LRSAINKTGTDEGALTRIVTTRAEIDLKVIGEEYQRRNSIPLEKAITKDTRGDYEKMLVALLGEDDA.
BnaC08g06690D   LRSAINKIGTNEGALTRIVTTRAEIDLKVIGEEYQRRNSIPLEKAITKDTRGDYEKMLVALLGEDDA.
BnaC05g27530D   LRSAINKTGTDEGALTRIVTTRAEIDLKVIGEEYQRRNSIPLEKAITKDTRGDYEKMLVALLGEDDA.
BnaAnng04520D   LRSAINKTGTDEGALTRIVTTRAEIDLKVIGEEYQRRNSIPLEKAITKDTRGDYEKMLVALLGEDDA.
Bo5g083900      LRSAINKTGTDEGALTRIVTTRAEIDLKVIGEEYQRRNSIPLEKAITKDTRGDYEKMLVALLGEDDA.
Bra034402       LRSAINKTGTDEGALTRIVTTRAEIDLKVIGEEYQRRNSIPLEKAITKDTRGDYEKMLVALLGEDDA.
Bo8g025760      LRSAINKIGTNEGALTRIVTTRAEIDLKVIGEEYQRRNSIPLEKAITKDTRGDYEKMLVALLGEDDA.
Bra039578       LRSAIKKTGTDEGALTRIVTTRAEIDLKVIGQEYQRRNSIPLEKIVNS....................
Bo6g043900      LRSAINKTGTDEGALTRIVTTRAEIDLKVIGQEYQRRNSIPLEKAITKDTRGDYEKMLIALLGEDDA.
BnaC06g08410D   ....................................................................
AT5G10230       LRQAINKLGTDEWGLTRVVTTRAEFDMERIKEEYIRRNSVPLDRAIAKDTHGDYEDILLALLGHDHA.
BnaA10g22010D   LRQAINKMGTDEWGLTRVVTTRAEFDMERIKEEYLRRNSVPLDRAIAKDTHGDYEDILLALIGHGHA.
Bra009048       LRQAINKMGTDEWGLTRVVTTRAELDMERIKEEYLRRNSVPLDRAIAKDTHGDYEDILLALIGHGHA.
BnaC09g46400D   LRQAINKMGIN.........................................................
Bo9g172330      LRQAINKMGIN.........................................................
BnaAnng37420D   LRLAINKMGTDEWALTRVVTTRTEVDMERIKEEYQRRNSIPLHHAVAKDTSGDYEDMLVSLLGHGDV.
BnaC02g43450D   LRLAINKMGTDEWALTRVVTTRTEVDMERIKEEYQRRNSIPLHHAVAKDTSGDYEDMLVSLLGHGDV.
Bra031890       LRLAINKMGTDEWALTRVVTTRTEVDMERIKEEYQRRNSIPLHHAVAKDTSGDYEDMLVSLLGHGDV.
Bo2g166530      LRLAINKMGTDEWALTRVVTTRTEVDMERIKEEYQRRNSIPLHHAVAKDTSGDYEDMLVSLLGHGDV.
BnaC03g49290D   LRLAINKMGTDEWGLTRVVTTRTEVDMERIKEEYQRRNSVPLDRAVAKDTSGDYEDMLVALLGHGDV.
Bra024346       LRLAINKMGTDEWGLTRVVTTRTEVDMERIKEEYQRRNSVPLDRAVAKDTSGDYEDMLVALLGHGDV.
BnaA06g23960D   ....................................................................
AT5G65020       LRLSINKMGTDEWGLTRVVTTRTEVDMERIKEEYQRRNSIPLDRAIAKDTSGDYEDMLVALLGHGDA.
AT5G10220       LRRAINRMGTDEWALTRVVTTRAEVDLERIKEEYLRRNSVPLDRAIANDTSGDYKDMLLALLGHDHA.
BnaC09g46410D   LRRAINKVGTDEWALTRVVTTRAEVDMERIKEEYLRRNSVPLDRAIANDTSGDYKDMLLALLGHDHS.
BnaA10g22020D   LRRAINKVGTDEWALTRVVTTRAEVDMERIKEEYLRRNSVPLDRAIANDTSGDYKDMLLALLGHDHS.
Bra009049       LRRAINKVGTDEWALTRVVTTRAEVDMERIKEEYLRRNSVPLDRAIANDTSGDYKDMLLALLGHDHS.
Bo9g172340      LRRAINKVGTDEWALTRVVTTRAEVDMERIKEEYLRRNSVPLDRAIANDTSGDYKDMLLALLGHDHS.
BnaC01g16910D   LRNAINTVGTDEDALTRVIISRAEKDLKKTTEPYHKRNNESLDQAIAKKTSRDYKAFLLALLGNGGI.
Bo1g039570      LRNAINTVGTDEDALTRVIVSRADKDLKKTTEPYHKRNNESLDQAIAKKTSRDYKAFLLALLGNGGI.
AT1G68090       LRKSMKGLGTDDTALIRIVVTRAEVDMQFIITEYRKRYKKTLYNAVHSDTTSHYRTFLLSLLGPNV..
BnaA02g13560D   LRKSVKGLGTDDTALIRMVVTRAEVDMHYIVSEYRKRYKKTLYNAVQSDTSGHYKTFLLSLLGPNV..
BnaC02g45910D   LRKSMKGLGTDDTALIRMVVTRAEVDMHYIVSEYRKRYKKTLYNAVQSDTSGHYKTFLLSLLGPNV..
Bra033961       LRKSVKGLGTDDTALIRMVVTRAEVDMHYIVSEYRKRYKKTLYNAVQSDTSGHYKTFLLSLLGPNV..
Bo2g057430      LRKSMKGLGTDDTALIRMVVTRAEVDMHYIVSEYRKRYKKTLYNAVQSDTSGHYKTFLLSLLGPNV..
AT2G38750       .DADKTTKKWLTRVFVTRADHSDEMNEIKEE..YNNLYGETLAQRIQEKIKGNYRDFLLTLLSKSD..
AT2G38760       VRDSIEGFGTDEDSLTRAIVTRAEIDLMKVRGEYFNMYNTSMDNAITGDISGDYKDFIITLLGSKI..
BnaC04g45920D   VRDSIEGFGTDEDSLTRGIVTRAEIDLMKARGEYFNMYNTSMDNAIIGDVSGDYKDFLLTLLGSNI..
BnaA04g22190D   VRDSIEGFGTDEDSLTRGIVTRAEVDLMKARGEYFNMYNTSMDNAIIDDVSGDYKDFLLTLLGSNI..
Bra017102       VRDSIEGFGTDEDSLTRGIVTRAEIDLMKARGEYFNMYNTSMDNAIIGDVSGDYKDFLLTLLGSNI..
Bo4g187790      VRDSIEGFGTDEDSLTRGIVTRAEIDLMKARGEYFNMYNTSMDNAIIGDVSGDYKDFLLTLLGSNI..
BnaA03g18080D   VRDSIEGFGTDEDSLTRAIVMRAEIDLMKVRGEYFNMYNASMDNAITGDVSGDYKDFLMTLLGSKI..
Bra000091       VRDSIEGFGTDEDSLTRAIVMRAEIDLMKVRGEYFNMYNASMDNAIIGDVSGDYKDFLMTLLGSKI..
BnaC03g21600D   VRDSIEGFGTDEDSLTRAIVMRAEIDLMKVRGEYFNMYNASMDNAIIGDVSGDYKDFLMTLLGSKI..
Bo3g032770      VRDSIEGFGTDEDSLTRAIVMRAEIDLMKVRGEYFNMYNASMDNAIIGDVSGDYKDFLMTLLGSKI..
BnaA03g18070D   SLNKDADKGTKKWLTRVFVTRADHSDMKEIAEEYNQLYGEPLAQTIQEKIKGNYRDFLLTLLSKSN..
BnaC03g21590D   SLNKDADKGTKKWLTRVFVTRADHGDMKEIAEEYNQLYGEPLAQTIQEKIKGNYRDFLLTLLSKSN..
Bra000090       SLNKDADKGTKKWLTRVFVTRADHSDMKEIAEEYNQLYGEPLAQTIQEKIKGNYRDFLLTLLSKSN..
Bo3g032760      SLNKDADKGTKKWLTRVFVTRADHGDMKEIAEEYNQLYGEPLAQTIQEKIKGNYRDFLLTLLSKSN..
BnaA04g22180D   .DADKGTKKWLTRVFVTRADHSDEMKEIAEE..YNQRYGETLAQNIQEKIRGNYRDFLLTLLSKSN..
BnaC04g45910D   .DADKGTKKWLTRVFVTRADHSDEMKDIAEE..YNQRYGETLAQNIQEKIRGNYRDFLLTLLSKSN..
Bra017103       .DADKGTKKWLTRVFVTRADHSDEMKEIAEE..YNQRYGETLAQNIQEKIRGNYRDFLLTLLSKSN..
Bo4g187780      .DADKGTKKWLTRVFVTRADHSDEMKDIAEE..YNQRYGETLAQNIQEKIRGNYRDFLLTLLSKSN..
```
