# Supplementary figures and images for: Comprehensive analyses of the annexin (ANN) gene family in Brassica rapa, Brassica oleracea and Brassica napus reveals their roles in stress response
Source: Sci Rep. 2020 Mar 9;10:4295. doi: 10.1038/s41598-020-59953-w (PMC7062692; doi:10.1038/s41598-020-59953-w)

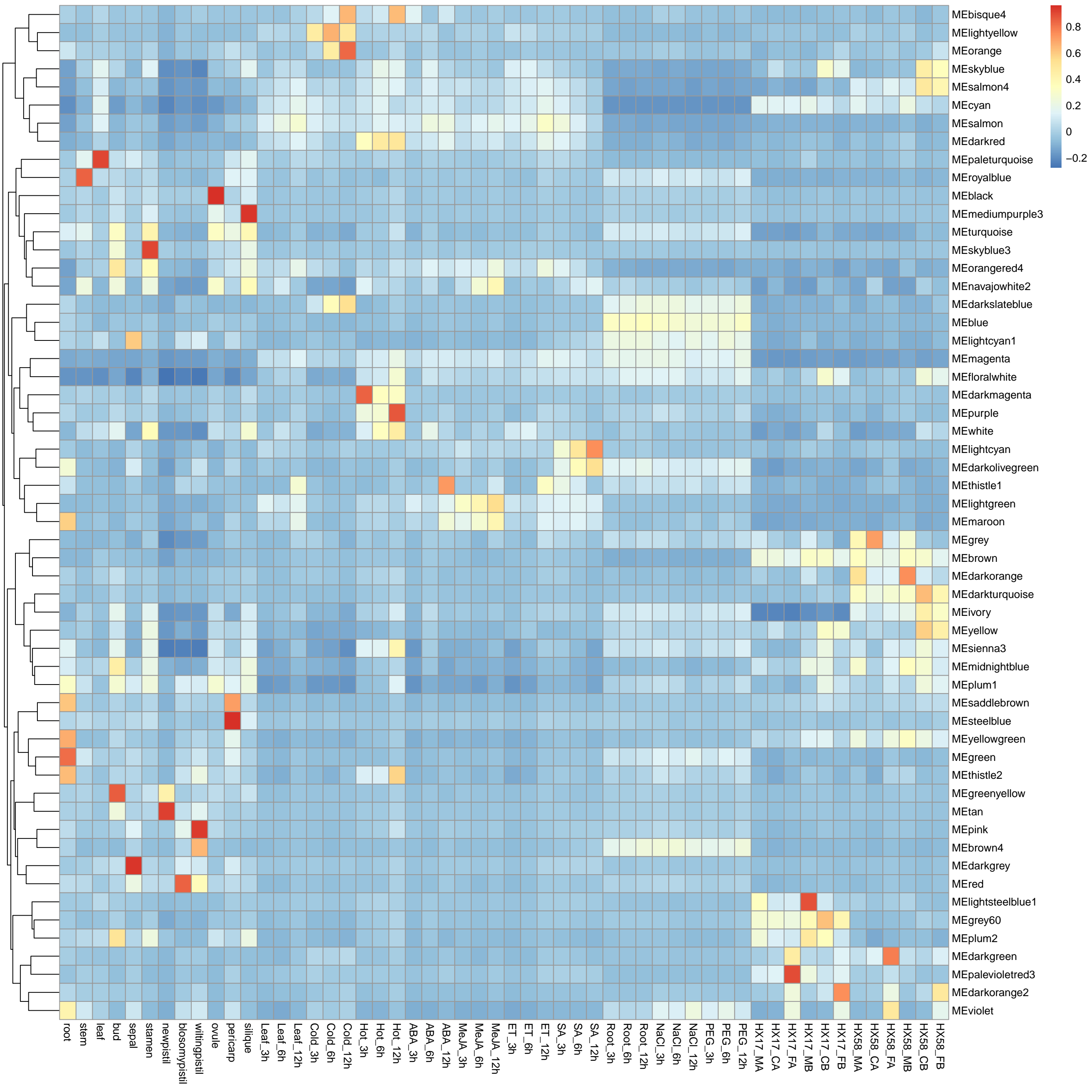

Supplement: Supplementary file 3 — Supplementary information 3. [file 41598_2020_59953_MOESM3_ESM.pdf]
