# Supplementary material for: Comprehensive analyses of the annexin (ANN) gene family in Brassica rapa, Brassica oleracea and Brassica napus reveals their roles in stress response
Source: Sci Rep. 2020 Mar 9;10:4295. doi: 10.1038/s41598-020-59953-w (PMC7062692; doi:10.1038/s41598-020-59953-w)
Supplement: Supplementary file 4 — Supplementary information 4. [file 41598_2020_59953_MOESM4_ESM.pdf]

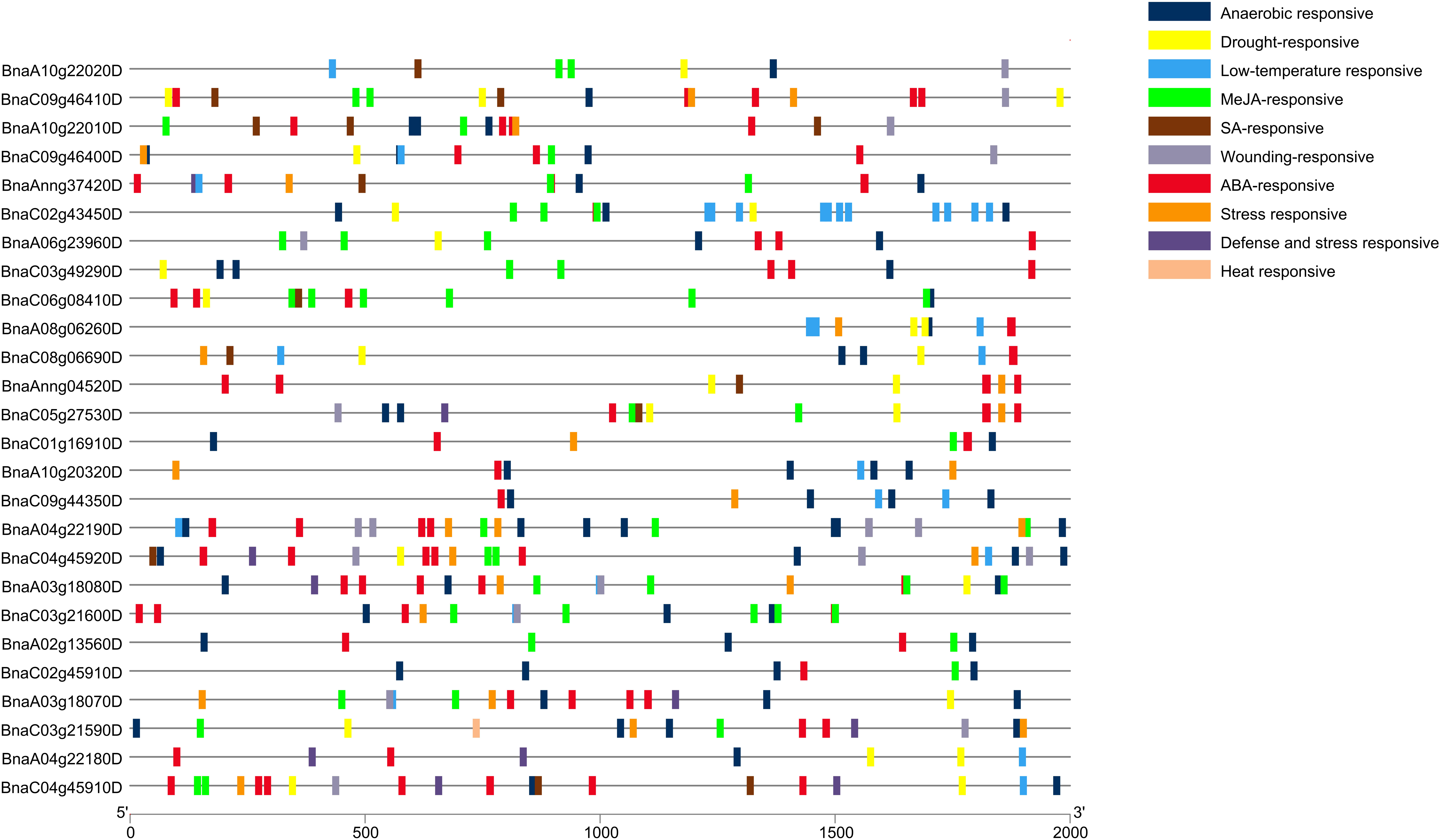

BnaA10g22020D
BnaC09g46410D
BnaA10g22010D
BnaC09g46400D
BnaAnng37420D
BnaC02g43450D
BnaA06g23960D
BnaC03g49290D
BnaC06g08410D
BnaA08g06260D
BnaC08g06690D
BnaAnng04520D
BnaC05g27530D
BnaC01g16910D
BnaA10g20320D
BnaC09g44350D
BnaA04g22190D
BnaC04g45920D
BnaA03g18080D
BnaC03g21600D
BnaA02g13560D
BnaC02g45910D
BnaA03g18070D
BnaC03g21590D
BnaA04g22180D
BnaC04g45910D
5'
3'
0
500
1000
1500
2000
Anaerobic responsive
Drought-responsive
Low-temperature responsive
MeJA-responsive
SA-responsive
Wounding-responsive
ABA-responsive
Stress responsive
Defense and stress responsive
Heat responsive
